# Predicting individual perceptual scent impression from imbalanced dataset using mass spectrum of odorant molecules

**DOI:** 10.1038/s41598-022-07802-3

**Published:** 2022-03-08

**Authors:** Tanoy Debnath, Takamichi Nakamoto

**Affiliations:** 1 Department of Information and Communications Engineering, Tokyo Institute of Technology, Tokyo, Japan; 2 Laboratory for Future Interdisciplinary Research in Science and Technology, Tokyo Institute of Technology, Tokyo, Japan

**Keywords:** Computational models, Machine learning, Cheminformatics, Olfactory system

## Abstract

Predicting odor impression is considered an important step towards measuring the quality of scent in the food, perfume, and cosmetic industries. In odor impression identification and classification, the main target is to predict scent impression while identifying non-target odor impressions are less significant. However, the effectiveness of predictive models depends on the quality of data distribution. Since it is difficult to collect large scale sensory data to create an evenly distributed positive (target odor) and negative (non-target odor) samples, a method is necessary to predict the individual characteristics of scent according to the number of positive samples. Moreover, it is required to predict large number of individual odor impressions from such kind of imbalanced dataset. In this study, we used mass spectrum of flavor molecules and their corresponding odor impressions which have a very disproportioned ratio of positive and negative samples. Thus, we used One-class Classification Support Vector Machine (OCSVM) and Cost-Sensitive MLP (CSMLP) to precisely classify target scent impression. Our experimental results show satisfactory performance in terms of AUC_ROC_ to detect the olfactory impressions of 89 odor descriptors from the mass spectra of flavor molecules.

## Introduction

Odor impression prediction is an active area of research that is important for evaluating product quality^1^. Human sense of smell is a complex process than visual and auditory perception^2^ as one odorant molecule can be described with more than two odor descriptors which is influenced by a person’s cultural background^3^ and experience. Odor is generally a complex mixture of many mono-molecular molecules that attaches and activates the olfactory receptors (ORs) of an Olfactory Sensory Neuron (OSN)^4,5^. Odors then stimulate our nasal olfactory neurons and the olfactory bulbs, thus converting these olfactory signals into odor impressions, such as ‘fruity’, ‘citrus’, ‘spicy’ etc.

Machine learning techniques have made significant progress in predicting odor impressions using molecular structure parameters^6^, activation information of the olfactory bulb^7^. Sanchez-Lengeling et. al^8^ used graph neural network to predict odor descriptors using a molecular graph structure. Another study had shown a report for predicting natural language descriptions of mono-molecular odorants using odor wheel^9^**.** Recently, Deepak et al. predicted smell impressions of ‘sweet’ and ‘musky’ using molecular structure parameters^10^**.** Odor, however, is usually a complex mixture, so it is better to establish a general method where we can use the same chemical features as the inputs of the machine learning model regardless of mixture or single molecule. The mass spectrum, an analytical technique that ionizes chemicals and sorts the ions based on their mass-to-charge ratio (m/z), can be used as input to the neural network model as it can be collected for both mono-monomolecular chemicals or chemical mixtures.

We used small mass spectrum dataset of mono-molecular chemicals, including continuous^11^ sensory data from the Dravnieks^12^ to predict the odor impressions. Then, relatively large mass spectrum dataset was used with binary form of odor descriptors from Sigma-Aldrich catalog^13^, which appears mutually exclusive, to predict the odor character of chemical using the natural language processing technique^14^. However, these studies^9,14–16^ focused on predicting odorant impressions by clustering similar smell impressions. Therefore, individual perception of odors is necessary for odor molecules to describe them.

Moreover, the problem of imbalanced ratio of negative to positive samples, which frequently appears in odor-impression prediction, deteriorates the prediction accuracy. We tried to solve this problem using oversampling technique^16,17^ where a part of the data was artificially generated. Although it improved the prediction accuracy to some extent, the improvement was limited because we could use only restricted artificial data. Creating artificial samples can duplicate samples from the minority class and this increases the likelihood of overfitting especially for high oversampling rates when class skew was severe^19^. The task of odor prediction is often imbalanced due to the presence of over and under-represented odor descriptors. So, our goal is to establish how to make a classification using the original small number of positive samples. Here the positive sample means the target odor with specified odor descriptors (e.g., fruity, pine, etc.) which we would like to predict from non-target (negative) samples. Non-target sample does not have specified odor descriptor. In this study, we will use the positive/negative and target/non-target words interchangeably.

Several odor impressions (e.g., fruity, sweet) appear very often and make it easier for human participants to describe with these common words. Thus, the number of positive appearances of uncommon odor impressions (‘hazelnut’, ‘peach’) is small. If the number of target-odors is much smaller than non-target odor samples, machine learning model only learns non-target odor samples well which affects the overall predictive performance. One of the methods to handle these large numbers of negative samples with small target odors is to use one class classification where we do not need equal proportions of positive and negative samples^18,19^. One class classification, a well-known method that has been applied to many research themes such as outlier detection etc. However, it has never been employed in odor prediction task. We chose OCSVM because only small number of positive samples was available here. Another possibility is to give weighted cost the minority samples during the training process with the help of neural network models trained with weighted loss functions.

Therefore, in this study we would like to establish a method for predicting individual odor perception from the mass spectrum of odorant molecules using highly imbalanced odor descriptors datasets without creating artificial samples. In odor prediction task, the main goal is to predict positive samples (target odor like ‘fruity’, ‘pine’) regardless of its occurrence frequency. However, the same algorithm might not be useful when the ratio of negative samples to positive ones varied. In this work, we used two separate algorithms (one class classification Support Vector Machine^20,21^and Cost Sensitive MLP^22^) to predict 89 odor impressions where each odor impression is highly imbalanced and has different occurrence frequency. We experimentally divided these odor descriptors into three categories, ‘large’, ‘middle’ & ‘small’, depending on the number of positive samples. We investigated the experimental results to select the correct algorithm as a function of the number of positive (target odor) and negative (non-target odor) samples.

The main contribution of this paper is the use of one-class classification Support Vector Machine (OCSVM) and cost sensitive multilayer perceptron (CSMLP) to evaluate the prediction performance from a large number of negative samples depending on odor descriptor occurrence in the dataset. Moreover, we established a rule for selecting the correct algorithm based on the ratio of negative to positive samples. We report that it can achieve better sensitivity or in other words, obtain better performance in predicting target odor with small category odor descriptors. To the best of our knowledge, our proposed method is the first to establish odor prediction system depending on the odor descriptor occurrences.

## Materials and methods

### Flavor database descriptions

**Figure 1:**
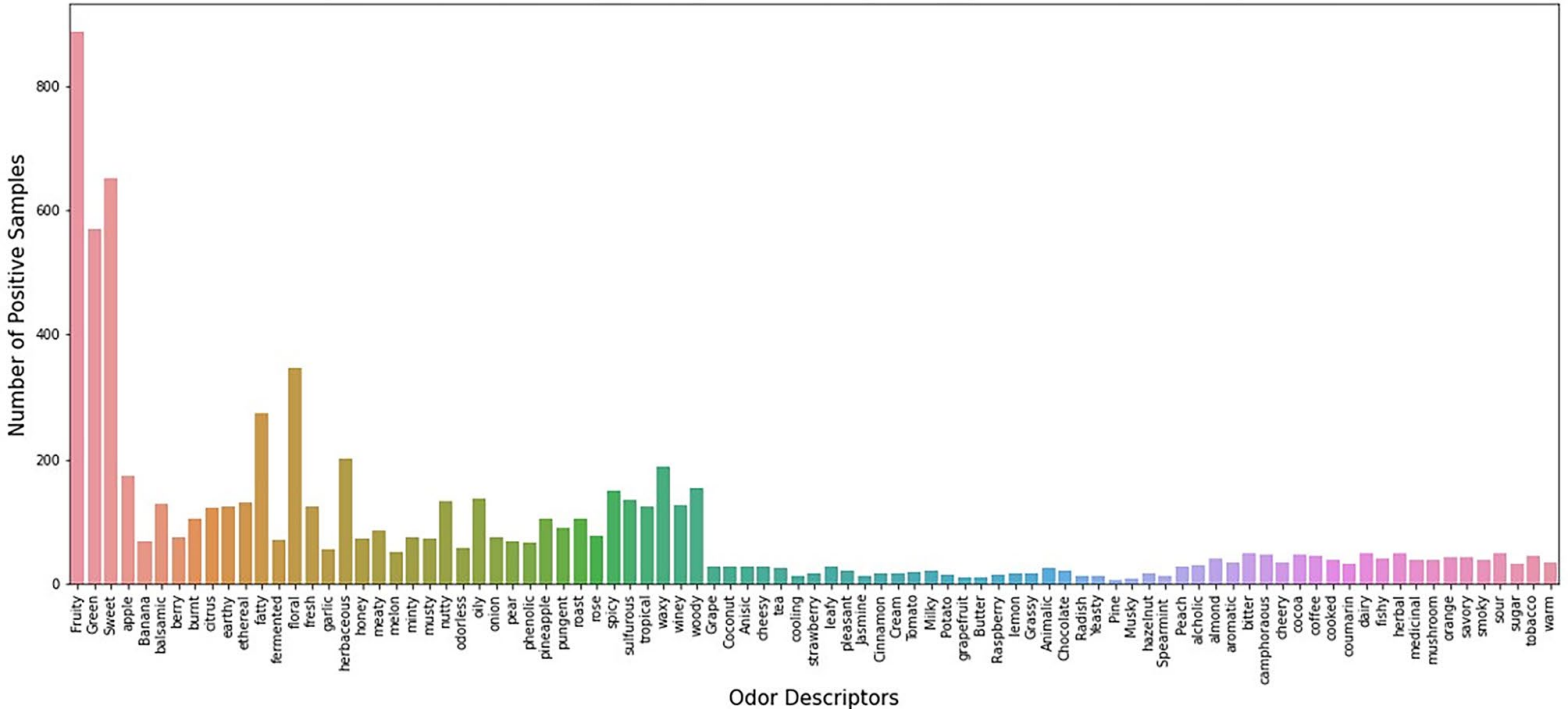
89 Odor descriptors with corresponding positive samples among 2345 odorant molecules.

Leffingwell^23^ Flavor Database (n = 2345) was used for this study where flavor molecules were labeled by one or more odor descriptors. The database contains chemical names with CAS number and their corresponding odor descriptions which are in free-form text. We obtained the mass spectrum of these flavor molecules from the Chemistry Webbook provided by National Institute of Standards and Technology (NIST)^24^ using the corresponding CAS number. Although original flavor database has more data, the verbal data without mass spectrum is eliminated. As one molecule is described with multiple odor descriptors, it creates a multi-label prediction problem. We try to solve it by decomposing the problem in several binary classification models. we got 89 odor descriptors including one odorless descriptor. We listed the name of odor descriptors and their frequencies of appearances among 2345 samples in Fig. 1.

### Data processing

Original mass spectrum of NIST has more than 300 dimensions. Intensity of 50–262 m/z (mass-to-charge ratio) was used because the intensity of m/z below 50 is mainly derived from odorless molecules like oxygen and intensity of m/z higher than 262 originates from molecules with low volatility. Thus, the data matrix is expressed by rows of 2345 samples and columns of 212 intensities. This data matrix of mass spectra was normalized in the range of 0 and 1 after dividing by the maximum value in the same mass spectrum. Principal component analysis (PCA) was used to reduce the dimensionality from these 212 intensities of mass spectrum. All the 2345 odorant molecules are listed with their CAS numbers in Supplementary file 1.

### Predictive model

#### One class classifier and binary SVM classifier

We used OCSVM to predict the target odor from mass spectrum of the imbalanced set of flavor molecules. OCSVM learns the task of making a decision boundary to classify new data as similar or different from the training set. The classifier tries to detect a single class and rejects the others. At first, the dataset was divided into majority (samples without target attribute) and minority class (samples with target attribute). Next, we created (train, test) tuples of majority samples for five-fold cross-validation. We split the majority class as we only have these majority samples during the training and minority class was added with the test split during the validation time. GridSearchCV (using Python libraries) was used to optimize the hyperparameters of OCSVM. We present the results for the model with the mean AUROC on each testing fold. We run the algorithm 89 times as we considered this analysis as a binary classification task of 89 odor descriptors.

We compare the performance of OCSVM with the binary SVM classifier using 5-fold stratified cross-validation where the only exception is that both positive and negative samples were used to train the binary SVM classifier.

#### Traditional & cost-sensitive multilayer perceptron

Two types of multilayer perceptrons were used for this study. One is traditional multilayer perceptron (MLP) and other is cost- sensitive multilayer perceptron (CSMLP). Traditional MLP trained by backpropagation of error algorithm considered misclassification costs (false negative and false positive) are the same, so a false negative is worse or more expensive than a false positive^25,26^. In the cost sensitive MLP, we assigned higher weight for the minority class and at the same time reduced the weight for the majority class. We determined the class-weights for the majority and minority classes in such a way that the model pays more attention to the observations from minority class. We scaled the weights of both classes so that the sum of the weights of all examples keeps the same, in other words, we assigned the class weights which is inversely proportional to their respective frequencies^27^.

5-Fold stratified cross-validation was used for evaluating both methods. The model was trained with 16 hidden neurons (empirically) in the 2^nd^ layer, Relu as the activation function and sigmoid at the final layer. Binary cross entropy was used as a loss function and model was trained with Adam^28^ (keras-optimizer) with learning rate 0.001. Drop out was used as a regularizer for preventing overfitting. The batch size was 64 and there was total 109 epochs. The difference between the two models was whether we determined the weight of class for was equal or not. The modified binary cross-entropy loss function that was used for CSMLP in Eq. (1).

1 $$\mathrm{weighted \,Binary\, crossentropy }=- \frac{1}{N} {\sum }_{i=1}^{N}[{w}_{0}\left({y}_{i}*\mathrm{log}(p({y}_{i}))+{w}_{1}((1-{y}_{i}\right)*\mathrm{log}\left(1-p\left({y}_{i}\right)\right)))]$$

where w_**j**_ = total samples / (n-classes * n-samples_**j**_).

Here, w_**j**_ is the weight for each class (j = 1: positive sample; j = 0: negative sample); total samples are the total number of samples or rows in the dataset; n-classes are the total number classes (in our case 2 class) in the target; n-samples_**j**_ is the total number of samples of the respective class.

## Results

### Principal component analysis

**Figure 2:**
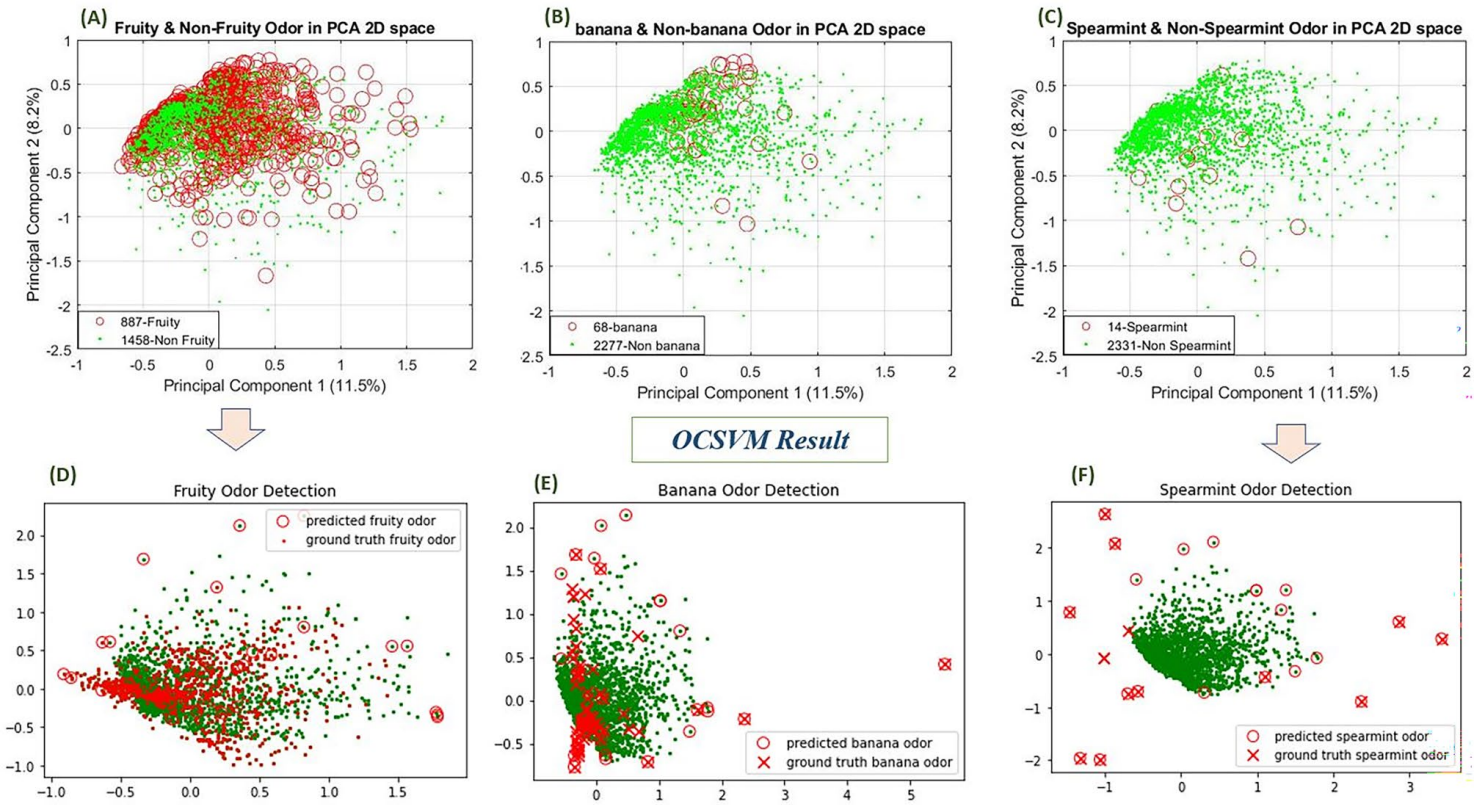
Top row, Principal component analysis in 2D space for each odor category. (**A**) Fruity (**B**) Banana & (**C**) Spearmint odor samples for large, middle & small category respectively. All are different figure although the location of each data point is the same. Red circle is different. Bottom row, (**D**), (**E**), (**F**) depicted the predicted vs ground truth odor detection for fruity, banana & spearmint using One class Support Vector Machine where green dots indicate the non-target odor samples for each example. Please see the supplementary file 2 for each odor descriptor detection using OCSVM for every category.

PCA was used to check the distribution of positive and negative samples for 89 odor descriptors as well as reducing the dimension to 25 optimally from its 212 intensities. We used 25 PCs for each odor descriptor in this study which capture more than 60% of the total variation. Increasing the number of PCs to more than 25 had no effect on the overall performance of the model. Figure 2 (top row) depicted scatter plot of the first two principal components (PCs) for fruity, banana & spearmint.

There was no clear separation between fruity and non-fruity samples as shown in 2D PCA plot Fig. 2A which indicates that samples are overlapping with one another and non-linear in structure also. Thus, the problem for discriminating its boundary gets complicated not only because of the data distribution but also for the skewness towards negative samples. For these types of samples where the data distribution overlaps, we can consider it as a large category odor descriptor as these odor descriptors appear most of the time among 2345 samples. There are totally 3 such kind of odor descriptors (fruity, sweet & green) in this study. The ratio of non-fruity to fruity, non-sweet to sweet and non-green to green are 1.64, 2.60 & 3.12 respectively in this experiment where the positive samples were around half of the negative ones. Such odor impressions can be predicted using MLP model by properly splitting the positive and negative samples during training period and using the suitable evaluation metric for describing the result of odor prediction of imbalanced dataset. We chose area under ROC curve as that curve keeps AUC high by scoring most of negatives very lower.

There are 34 more odor descriptors where the ratio of negative to positive samples was between 5 ~ 44, thus the problem to predict the target odor becomes difficult for increasing the number of negative samples compared to the large category samples. Figure 2B depicts the banana and non-banana odor samples in 2D space where target odor (banana) overlaps with non-banana samples. We can consider these kind of odor impressions as a middle-class odor descriptor.

However, the prediction gets more complicated when the ratio of negative to positive samples were too high (ratio of non-spearmint to spearmint is 166.5), for example spearmint (14 positive samples) was depicted as Fig. 2C. Such problems are difficult to solve due to the high skewness towards negative class (2331 negative samples). One hypothesis is to solve this problem by considering the minority samples as an outlier because most target odors are outside the dense boundary of negative samples. In this analysis, we found 51 odor descriptors where the number of positive samples was below 50 and, in all cases, these target odors were almost outside the decision boundary of non-odor samples. The ratio of negative to positive samples is between 46 ~ 334 for these 51 odor descriptors. We have renamed it as a small category positive sample.

### One class SVM & binary SVM classifier

The most important part of One class SVM was optimizing the hyperparameters kernel, gamma (σ), nu(ν). The number of support vectors decreases with σ increasing and the decision boundary becomes unclear. The parameter ν also affects the shape of decision boundary; as ν increases, the number of support vectors increases, and so does the number of incorrectly classified training samples increase. It is usually set to a small value to ensure a small misclassification rate on the training phase. During the optimization, we selected the range ν from 0.01 to 0.3, set the σ as scale or auto and used radial basis function kernel (RBF) as a kernel for optimizing these hyperparameters using scikit-learn One class SVM^29^. Surprisingly, the hyper-parameters optimized (ν = 0.01 and σ = auto) for one class SVM of 89 odor descriptors were the same.

One hypothesis for such results is the training dataset of negative (non- target odors) samples which is almost the same for all classifications for selecting the boundaries of these samples because their original distribution was the same. We will fit a distribution or decision boundary for the negative samples and then use the trained model to label the validation set to see if the given sample is positive or negative.

**Figure 3:**
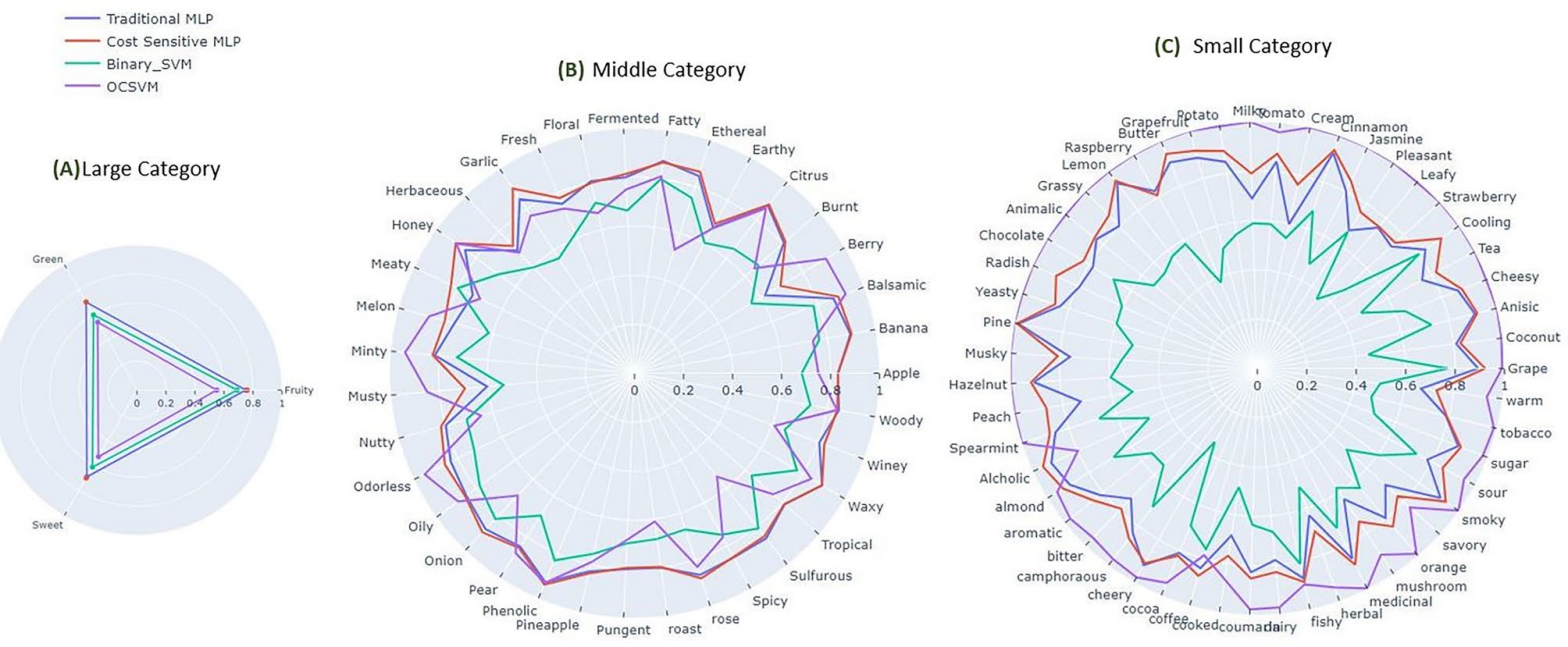
5-fold cross validation results to show AUC for each odor category; (**A**) large (**B**) Middle & (**C**) Small category odor samples_._ Blue, Red, Green & purple line depicted the result for Traditional MLP, cost sensitive MLP, Binary SVM and One class SVM respectively. We present the results for the model with the mean AUROC on each testing fold for five-fold cross-validation method.

The data distribution of large category odor descriptors (fruity, green, sweet) was complex and the ratio of negative to positive samples was not so high. The results of the fivefold cross validation (Mean AUC_ROC_) of these three descriptors are shown in Fig. 3A (purple). The Mean AUC_ROC_ for these three odor descriptors is very low (below 0.60). For example, the decision boundary made by the non-fruity samples during the training completely overlaps with the validation dataset that included both the non-fruity and fruity samples shown in Fig. 2D. These results indicate that it is not feasible to use one class classification when the ratio of negative to positive samples is small (between 1.5 ~ 4 approximately).

Odor classification becomes more difficult when the number of target class is too small, meaning the ratio of negative to positive samples is very high. Each of these 51 odors descriptors have less than 50 positive samples out of 2345. Thus, it is a problem to discriminate such positive samples (specific odor descriptor such as spearmint) from negative samples (non-spearmint) as shown in Fig. 2C. For example, 11 spearmint odors were clearly predicted, and other ground truth samples could not be accurately identified as shown in Fig. 2F. There were some errors with red circle that only indicated false detection of odors but note that such false detection occurs at the border of the boundary of negative (non-spearmint) class. Figure 3C (purple) is the result (Mean AUC_ROC_) of a classification of 51 odor descriptors. Traditional binary classification is not a suitable method to use in such cases because of the class skewness, thus the model only learns the negative class and does not generalize well for the unseen positive class. A good AUC_ROC_ score (more than 0.90) has been obtained for all of these odor descriptors using one class support vector machine except alcoholic, coffee (below 0.80). So, it is feasible to use OCSVM for identifying odor when its attribute appears infrequently.

When the number of positive classes is more than 50 in other words when the ratio of negative to positive samples increases compared to the small category samples, the results were not good as the previous small category samples. The decision boundary of positive and negative samples was difficult to discriminate due to overlapping between positive and negative samples. We found 35 odor descriptors with positive samples between 51 and 350. For example, banana samples (68 positive samples) were not accurately predicted because they were overlapping with non-banana (2277) samples as illustrated in Fig. 2E. Banana samples that were located within the decision boundary of the non-banana samples were very difficult to distinguish from the non-banana sample and this was true for the other 34 middle category odor descriptors. We got comparatively low AUC_ROC_ score (below 0.80) for apple, banana, burnt, earthy, ethereal, fermented, floral, fresh, garlic, herbaceous, meaty, nutty, onion, pineapple, pungent, roast, spicy, sulfurous, tropical, winey as shown in Fig. 3B (purple).

We used SVM to compare with OCSVM. We found a better performance (based on AUROC shown in green line of Fig. 3A) of SVM than OCSVM for the large category. Out of 35 odor descriptors in middle category, only 11(banana, burnt, ethereal, floral, meaty, nutty, onion, pungent, roast, sulfurous, winey) have better performance using SVM shown in Fig. 3B-green (see supplementary file 6-SVM column). However, none of the odor descriptors show good performance (Fig. 3C) for small category dataset. This is due to the small number of target samples. So, typical binary classification is not a good choice when the occurrence of the target (positive) samples is very small (below 50 in our case). We reported the optimal hyperparameters (C, and gamma) for binary SVM in supplementary file 3.

So, it can be said that we can use OCSVM when we have a very small number of target odors. In this type of small category samples where the positive samples are outside the decision boundary of the negative samples, it will be better to use OCSVM to accurately predict the target odor. On the other hand, it is not appropriate to use OCSVM for middle and large category data.

### Traditional and cost-sensitive multilayer perceptron

Since one class classification failed to detect the target odor samples when the number of positive samples was higher than middle and small category odor samples, we used cost-sensitive multilayer perceptron & compared them to traditional MLP.

We got a satisfactory Mean AUC_ROC_ (in both cases around 0.80 which is better than OCSVM) as shown in Fig. 3A (Blue & Red line for Traditional and Cost-sensitive MLP respectively) for these three descriptors using a 5-fold stratified cross-validation of these two methods. Mean AUC_ROC_ score has been slightly increased for these high category odor descriptors using Cost-sensitive MLP. Compared to the results of traditional (blue) & cost-sensitive (red) MLP shown in Fig. 3C, OCSVM (red) has shown better performance in predicting target odors from the large number of negative samples, in other words, when the number of positive samples was very small (< 50). OCSVM has reported the best prediction performance (> 0.90) for almost all odor descriptors. We got a relatively good AUC_ROC_ score (around 0.80) using CSMLP compared to OCSVM for each middle category odor descriptors except berry, earthy and musty, as shown in Fig. 3B. However, these three odor descriptors had lower prediction performance using traditional MLP, which was improved slightly using cost sensitive MLP.

However, this experiment will be clearer if we do the experiment using train-validation-test split method. Considering the low numerosity of data, we experimented this analysis by dividing the dataset into training, validation, and test sets. The model was trained and validated with 1899 & 211 samples respectively. Although initially we set the epoch 109, we stopped model training at the best validation error instead of a fixed number of epochs. The model was then tested on the 235 samples that included both target and non-target samples. Similar sets of training/validation/testing samples were used in both MLP and CSMLP cases. We have shown the results of large category odor descriptors in Table 1. We have provided supplementary file 4 for the middle and small category datasets.

**Table 1:** Traditional MLP (top) & cost sensitive MLP (bottom) result for predicting target odor (large category) for the testing set. TP = True Positive; TN = True Negative; FP = False Positive; FN = False Negative.

Large Category Odor Descriptors Traditional MLP									
Name of OD	TP	TN	FP	FN	ROC AUC	Recall	Target smell during testing		
Fruity	30	119	24	62	0.655	0.326	92		
Green	0	182	0	53	0.585	0.000	53		
Sweet	11	154	5	65	0.681	0.145	76		

Large Category Odor Descriptors Cost Sensitive MLP									
Name of OD	TP	TN	FP	FN	ROC AUC	Recall	Target smell during testing	Weight for class 0	Weight for class 1
Fruity	76	61	82	16	0.658	0.826	92	0.8	1.32
Green	28	119	63	25	0.591	0.528	53	0.66	2.06
Sweet	46	103	56	30	0.676	0.605	76	0.69	1.8

Table 1 shows the comparison between traditional (top) and cost sensitive MLP (bottom) to predict more true positive samples from the test sets. For example, there were total 92 fruity samples in testing set. Traditional MLP model identified only 30 true positive (fruity) samples, thus recall and the area under ROC curve was not so high. Cost sensitive MLP detected 76 true positive samples, in other words, class weights increased recall because the model found more true positives samples, thus decreasing the false negatives.

### Statistical analysis for OCSVM on small category dataset

We have further analyzed on the small category of odor descriptors because our claim is: ‘OCSVM is more precise for target odors with low occurrence of odor descriptors (51 odor descriptors used in our case)’. We have analyzed in reverse process. (1) First, we created (train, test) from positive samples for the five-fold cross validation; (2) While four-fold of positive samples were used for training, the remaining fold and negative samples were used together for testing each time. Thus, we got 51 pairs (first method where we used negative samples for OCSVM training and another one where we used positive samples for training) [see supplementary file 5].

A paired samples t-test (significance level was set at an alpha of *P* < 0.05) was conducted to compare the difference of training with positive samples and training with negative samples. There was a significant difference in the scores for training with positive samples (M = 0.607, Var = 0.0058) and training with negative samples (M = 0.970, Var = 0.0026) conditions; t (50) = − 23.22, P = $9.65\times {10}^{-29}$. These results suggest a statistically significant difference between OCSVM performance and odor descriptor numerosity (explained with training samples) without considering the data distribution.

**Figure 4:**
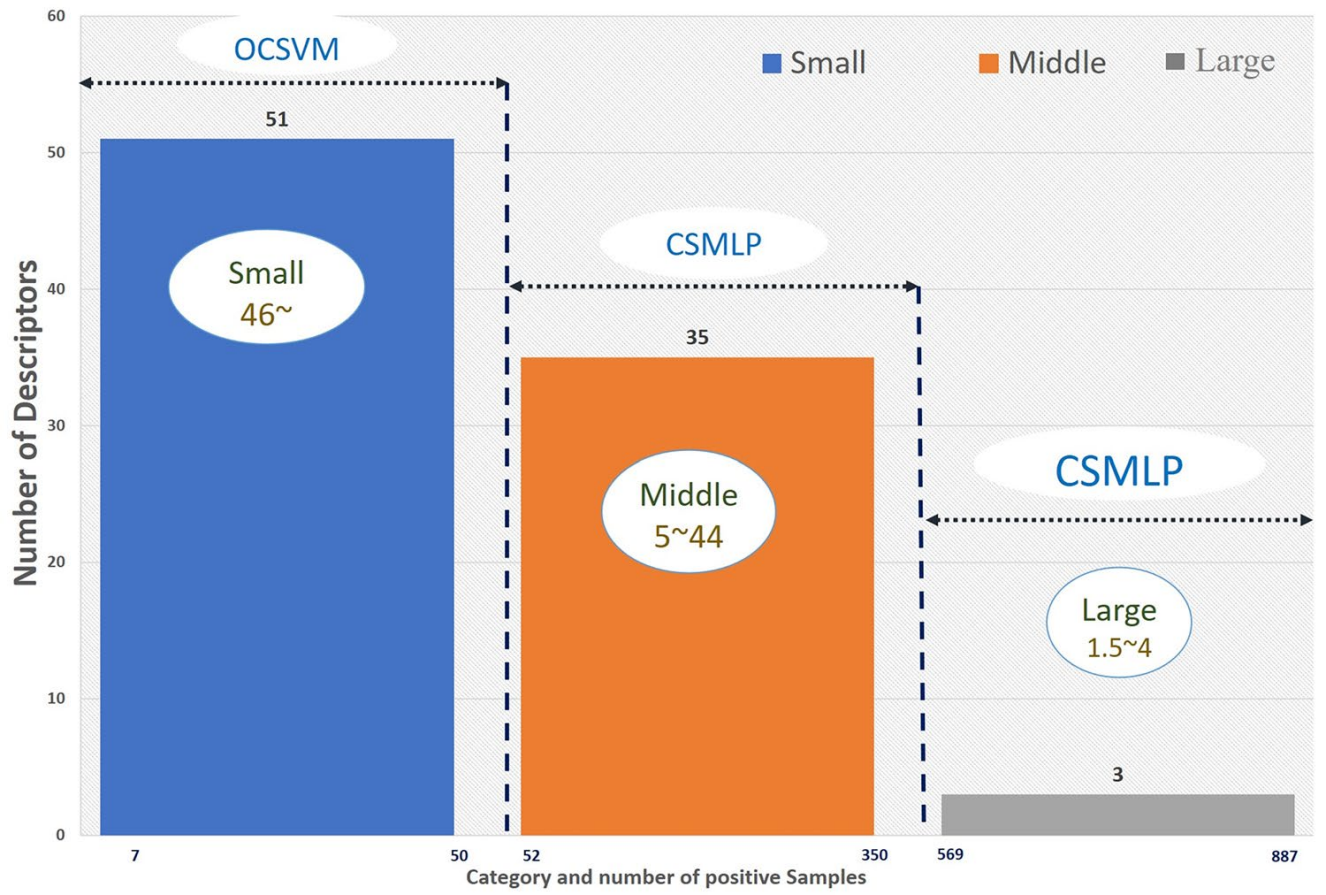
Horizontal axis shows the range of positive samples & vertical axis is the no. of odor descriptors of each category. Ratio of negative to positive samples are also shown here for each category (inside the box).

### Discussion

Finally, based on the above experiment we have made a decision for choosing the appropriate algorithm to predict the odor impression as a function of the number of positive samples as depicted in Fig.4. We found a large number of small category odor descriptors (no. of positive samples between 7 and 50) where the ratio of negative to positive was between 46 ~ 334 using the flavor database. In odor prediction tasks, the small number of target odors affects the overall performance of the classification. As the number of target odors is very small, one class classification is a better approach for such kind of situation. We can treat these small number of positive samples as an outlier, so it will be easier to predict the odor impression with these limited number of positive samples. On the other hand, 35 odor descriptors have been found in this study where the number of positive samples were between 52 to 346. Ratio of negative to positive samples were between 5 ~ 44. Experimental results showed that CSMLP gave better result to detect the true positive samples for most of the odor descriptors in this category compared to the one class SVM. However, when the ratio of negative to positive samples was between 1.5~4, in other words, number of positive samples was half of the negative samples, CSMLP showed better performance than OCSVM. Most recent work^8^ used Leffingwell database to predict odor perception using Graph neural network. We reported comparisons in supplementary file 6 for each odor descriptor that are common both in our study and in the paper^8^ that used molecular graph structures as inputs for the Graph neural network. Although this is completely different from our case because we used the mass spectrum as an input, we can compare OCSVM & CSMLP with GNN^8^, traditional MLP and normal SVM in terms of predicted performance of each odor descriptor. We present the results of the model with the mean AUROC in each test fold for five-fold cross-validation method. We noticed that 51 (43 & 8 from small & middle category respectively) odor descriptors (marked in red color in supplementary File 6) showed better AUROC performance in our approach. Most of the odor descriptors are from small category where number of positive samples is very small. Based on the supplementary file 6, we did a paired samples t-test (significance level was set at an alpha of *P* < 0.05) on small category odor descriptor’s result (47 odor descriptors are common in GNN result and our result (OCSVM). There was a significant difference in results between GNN (M = 0.892, Var = 0.004) & OCSVM (M = 0.968, Var = 0.003); t (46) = − 5.97429, P = $1.58\times {10}^{-7}$. Although GNN is slightly better than other methods in case of large and middle categories, OCSVM is much better than GNN in small category.

## Conclusion

Machine learning approaches have been used to predict the smell impression; however, previous studies did not use odor descriptors themselves but used odor descriptor groups to describe the scent. Since there are very few target samples for most of the odor descriptors, selecting an appropriate computational method that can address this limitation for the odor prediction task can be considered as the solution with most reliability.

In this work, we propose OCSVM as a one-class classification method, and Cost-sensitive MLP to classify and predict target odors from a large number of negative (non-odor) samples using mass spectrum of odorant molecules. The main goal of one-class classifier is to separate positive samples from others, we use it to find target odors (positive set) that has the similar objective. In this specific problem, the target is to detect odors while identifying non-target samples are of less or no significant.

We have divided the odor descriptors into three categories according to their presence in the odorant molecules so that the over-represented and under-represented odor descriptors are clearly understood. Since such under-represented (peach, pine, etc.) odor impressions are large (in our case 51), we should keep good accuracy for under-represented odor descriptors.. GNN model^8^ can perform well in large (only 3%) and medium (39%) category descriptors, but we have still 57% small category descriptors that we need to predict. Experimental results suggest that OCSVM is suitable for use when the number of such odor descriptors is very small (less than 50 in our experiment). We also found a statistically significant difference between OCSVM performance and odor descriptor numerosity (explained with training samples) without considering the data distribution and result between previous study^8^ & OCSVM. So, our proposed method could be a way to predict odors for small category odor descriptors.

Moreover, traditional, and Cost-sensitive MLP was used for comparing the results with one class classification. Empirical results showed that cost sensitive MLP had better predictive performance than traditional one. It can be used for all the cases (large, middle & small). However, when the number of odor descriptors are very small, OCSVM is a better predictive model. So, the numerosity of a given odor descriptor is sufficient to select the method to be used (CSMLP or OCSVM).

The major limitation in our study is that our current study is limited to only Leffingwell database only. It can be extended to another available flavor dataset in the future. In conclusion, our study provided an idea that OCSVM and Cost-sensitive MLP could be useful for predicting scent impression, with a limited number of target samples and without generating the artificial observations for balancing the number of positive and negative samples.

## Supplementary Information

Supplementary Information 1.

Supplementary Information 2.

Supplementary Information 3.

Supplementary Information 4.

Supplementary Information 5.

Supplementary Information 6.
