# Supplementary material for: Predicting individual perceptual scent impression from imbalanced dataset using mass spectrum of odorant molecules
Source: Sci Rep. 2022 Mar 8;12:3778. doi: 10.1038/s41598-022-07802-3 (PMC8904784; doi:10.1038/s41598-022-07802-3)

Here, we depicted the predicted vs ground truth odor detection for each odor descriptor using OCSVM for every category.

**Figure S1:** Large Category odor descriptors (Green and Sweet) prediction


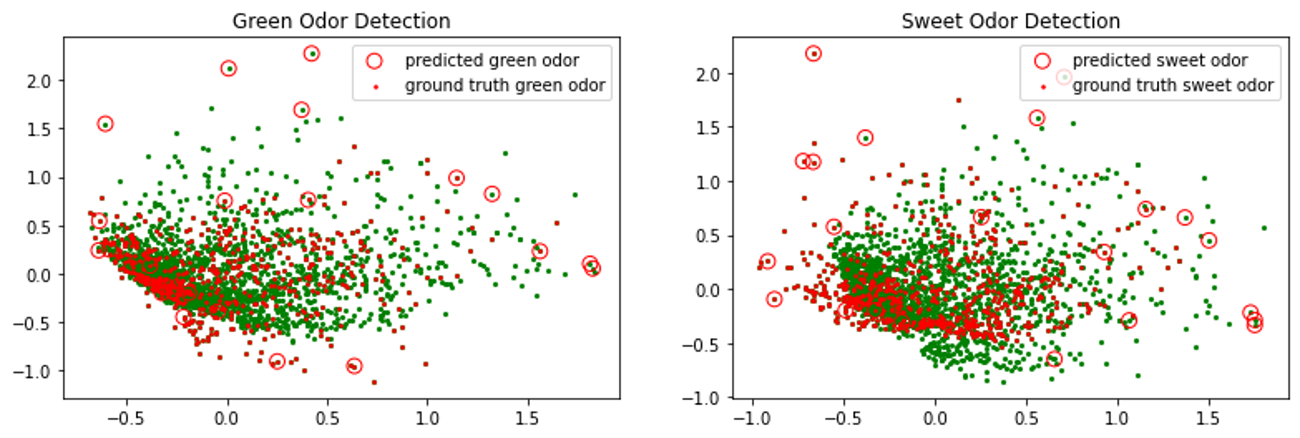


**Figure S2:** Middle Category odor descriptors prediction. Here we depicted apple, balsamic, berry, burnt, citrus, earthy, ethereal, fatty, fermented, floral, fresh, garlic, herbaceous, honey, meaty, melon, minty, musty, pear, onion, oily, odorless, nutty, phenolic, sulfurous, pineapple, pungent, spicy, roast, rose, waxy, tropical, winey, woody


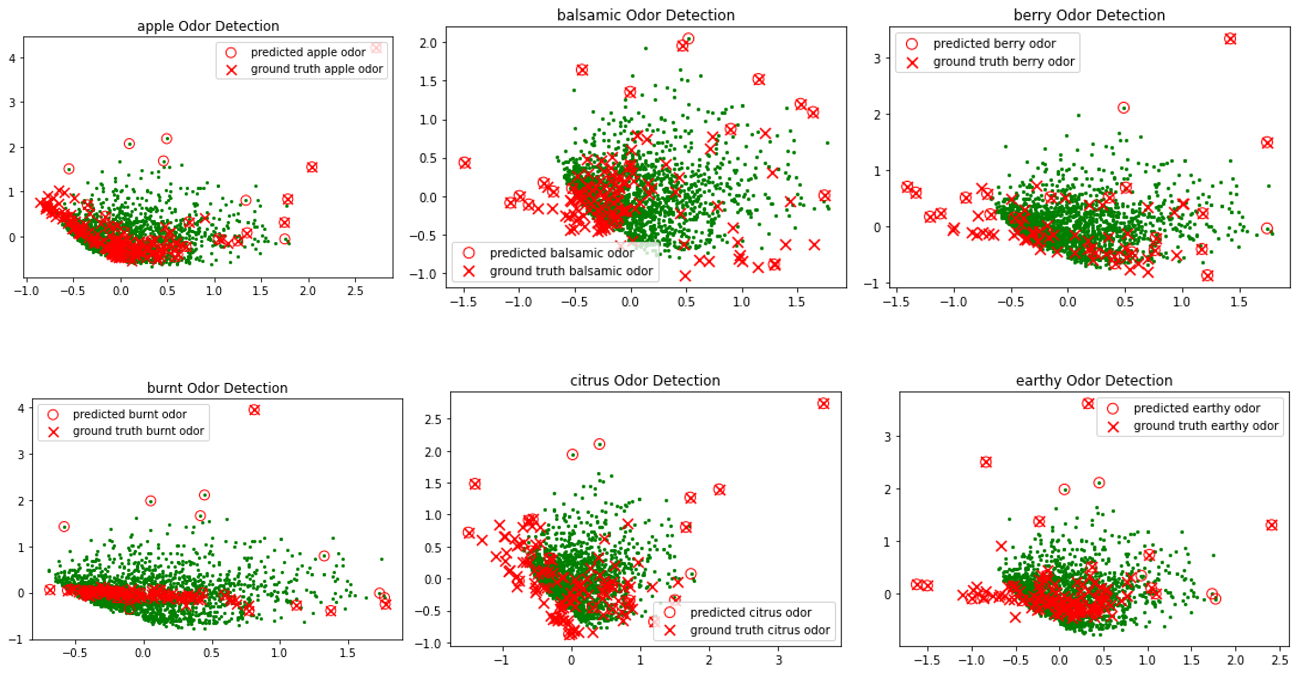


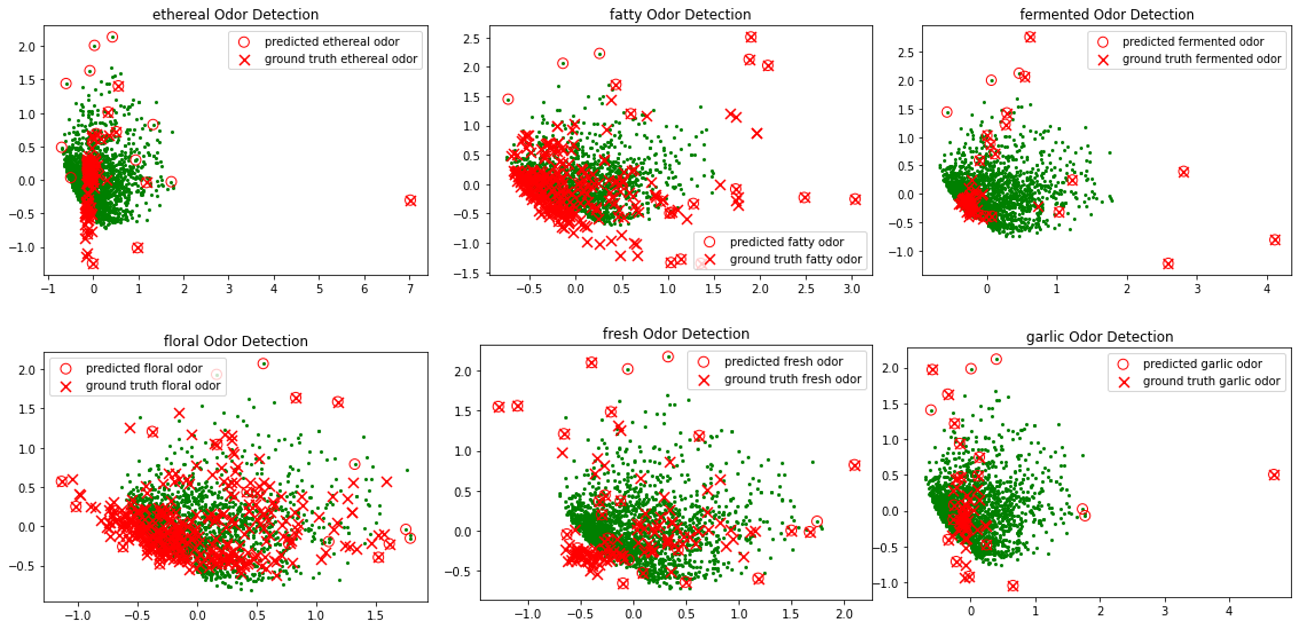


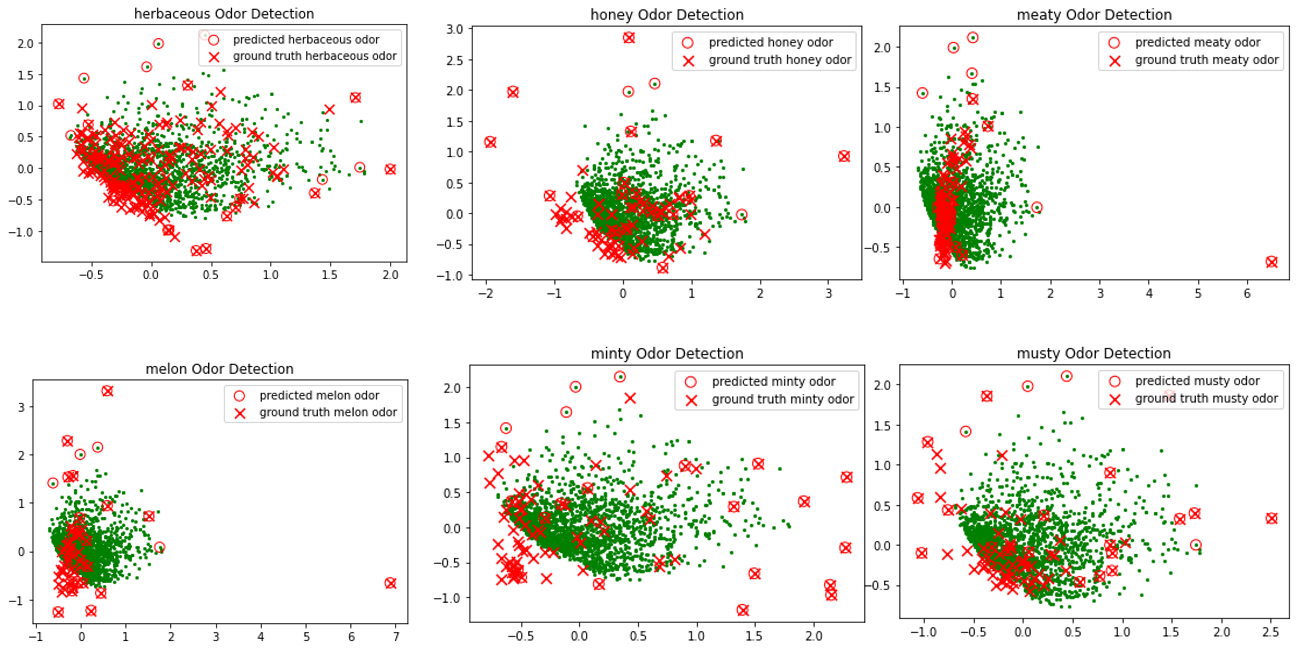


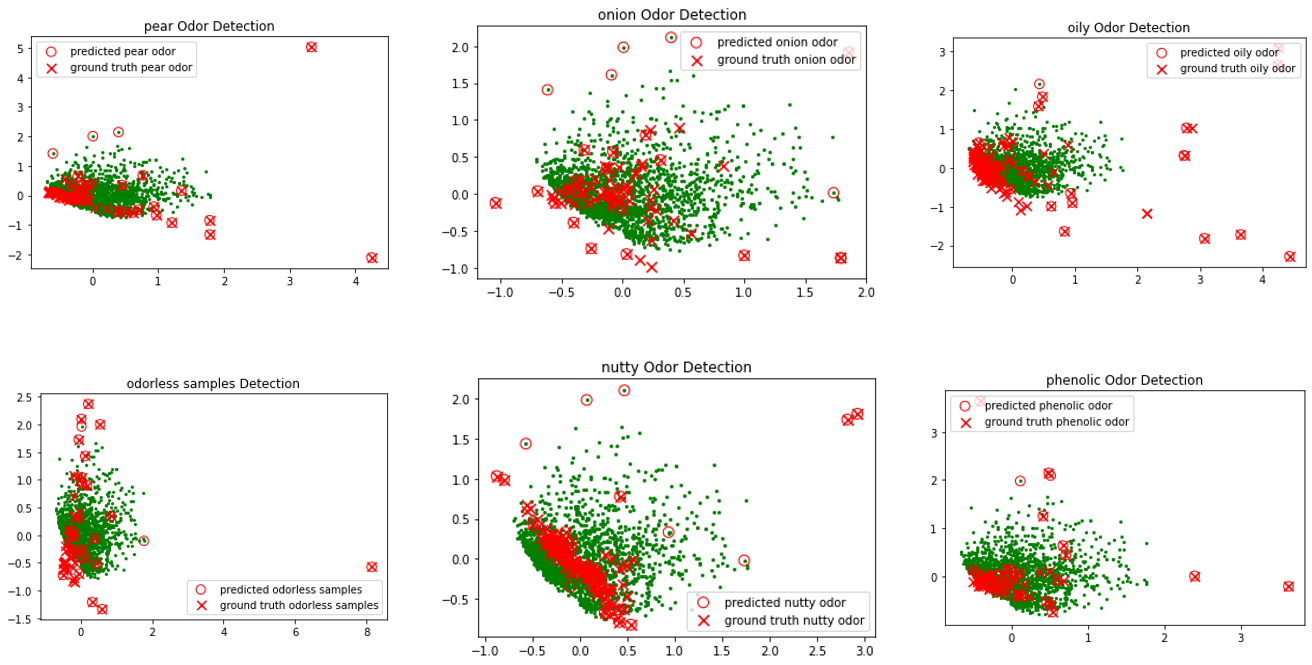


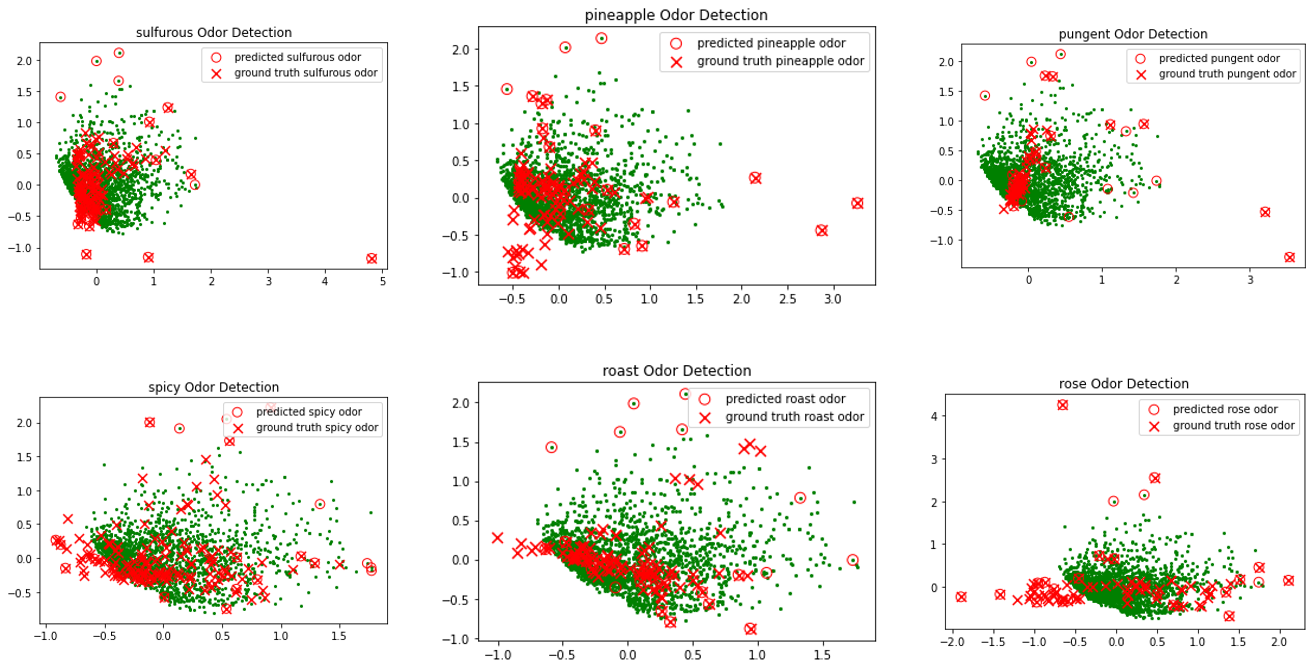


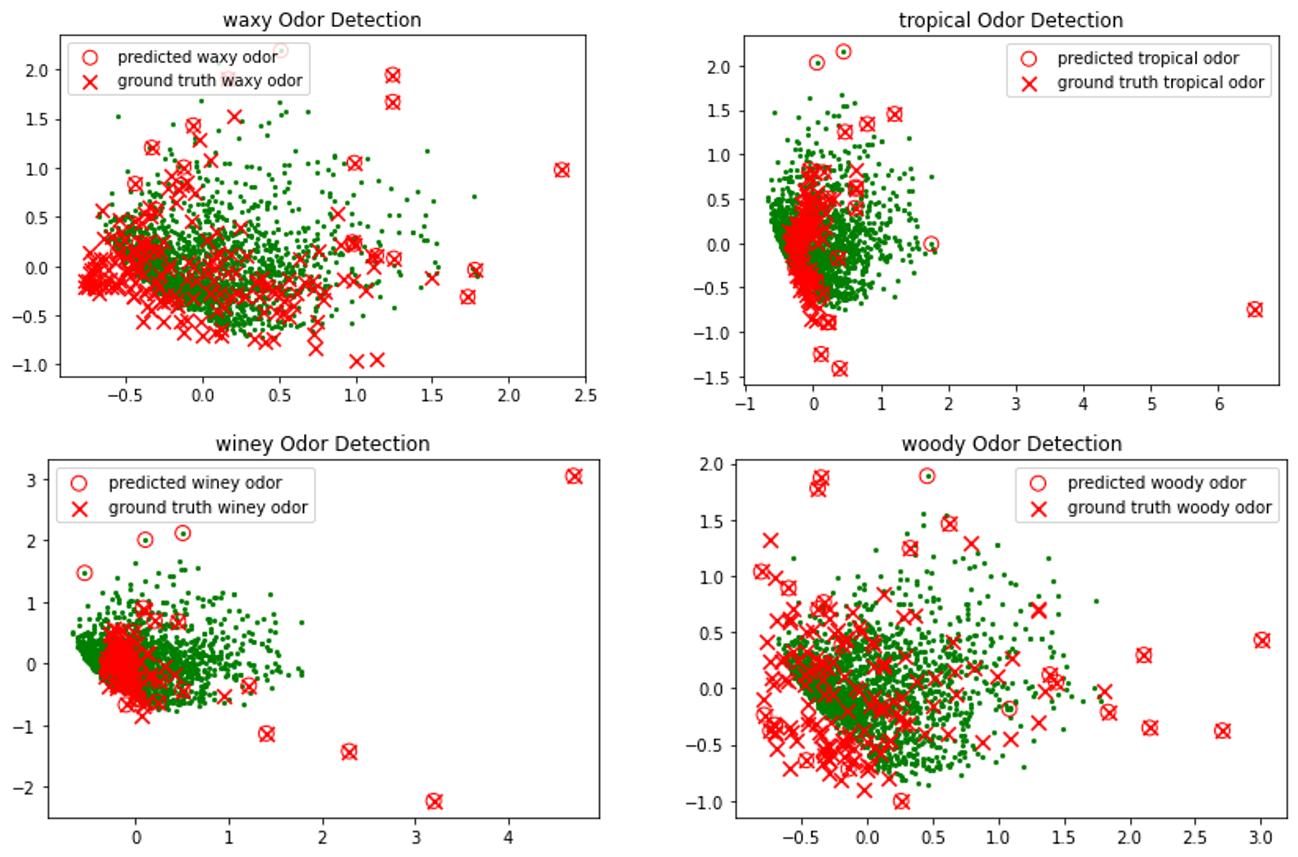


**Figure S3:** Small Category odor prediction. Here we depicted grape, coconut, anisic, cheesy, tea, cooling, strawberry, leafy, pleasant, jasmine, cinnamon, cream, tomato, milky, potato, grapefruit, butter, raspberry, lemon, grassy, animalic, chocolate, radish, yeasty, camphoraous, aromatic, bitter, alcoholic, cherry, almond, cooked, coffee, coumarin, dairy, cocoa, fishy, herbal, mushroom, orange, medicinal, hazelnut, musky, peach, savory, sour, smoky, sugar, pine, tobacco, warm.


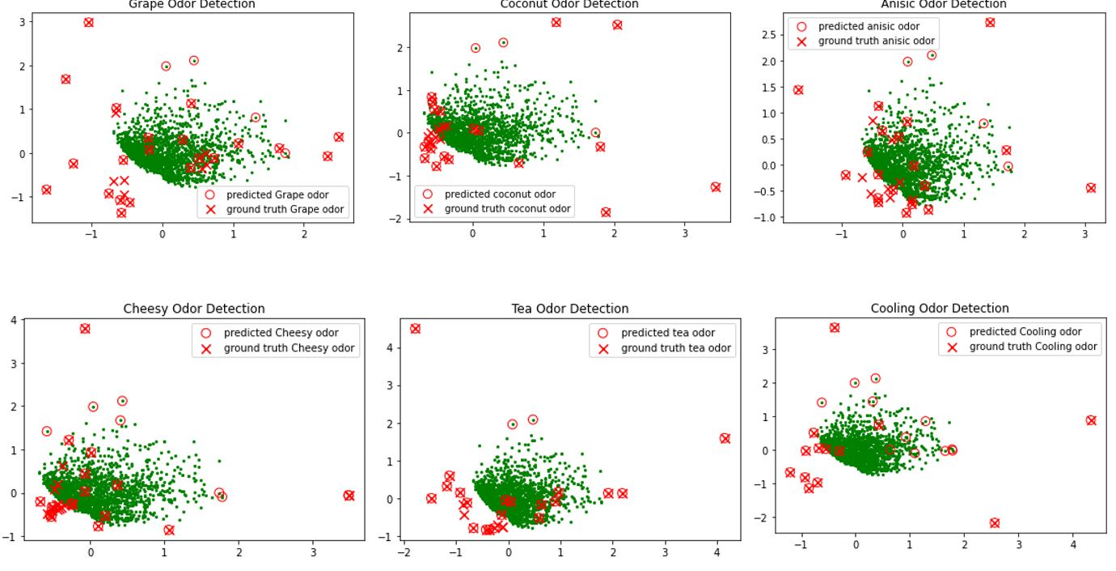


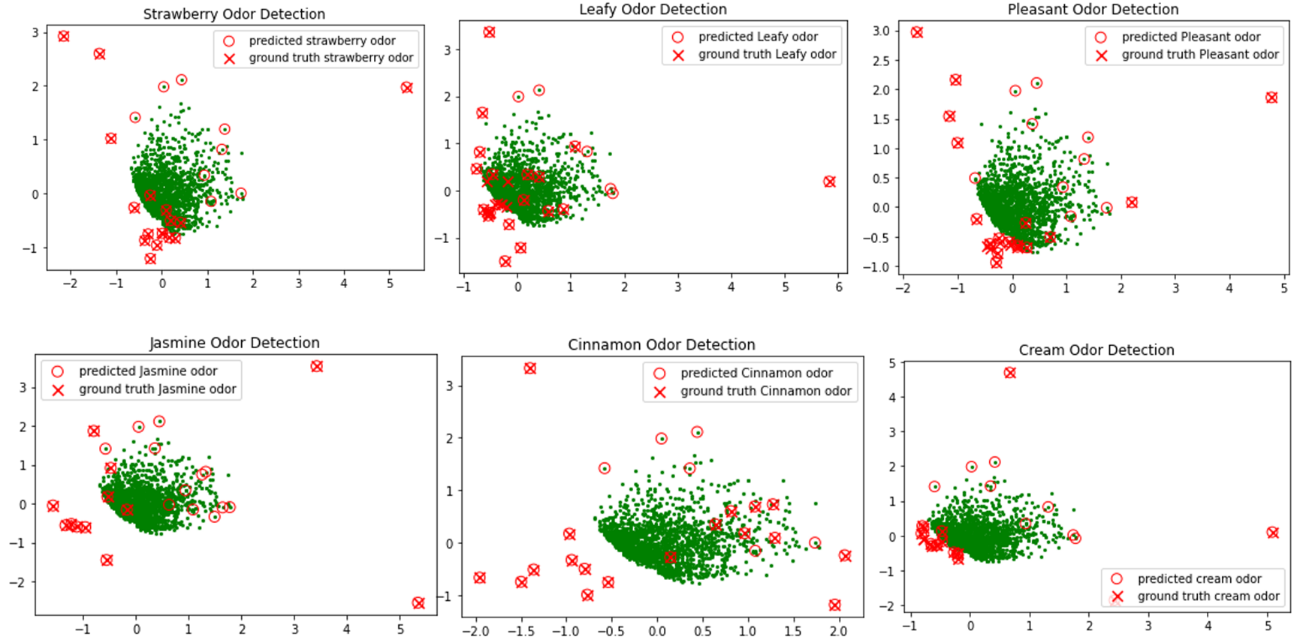


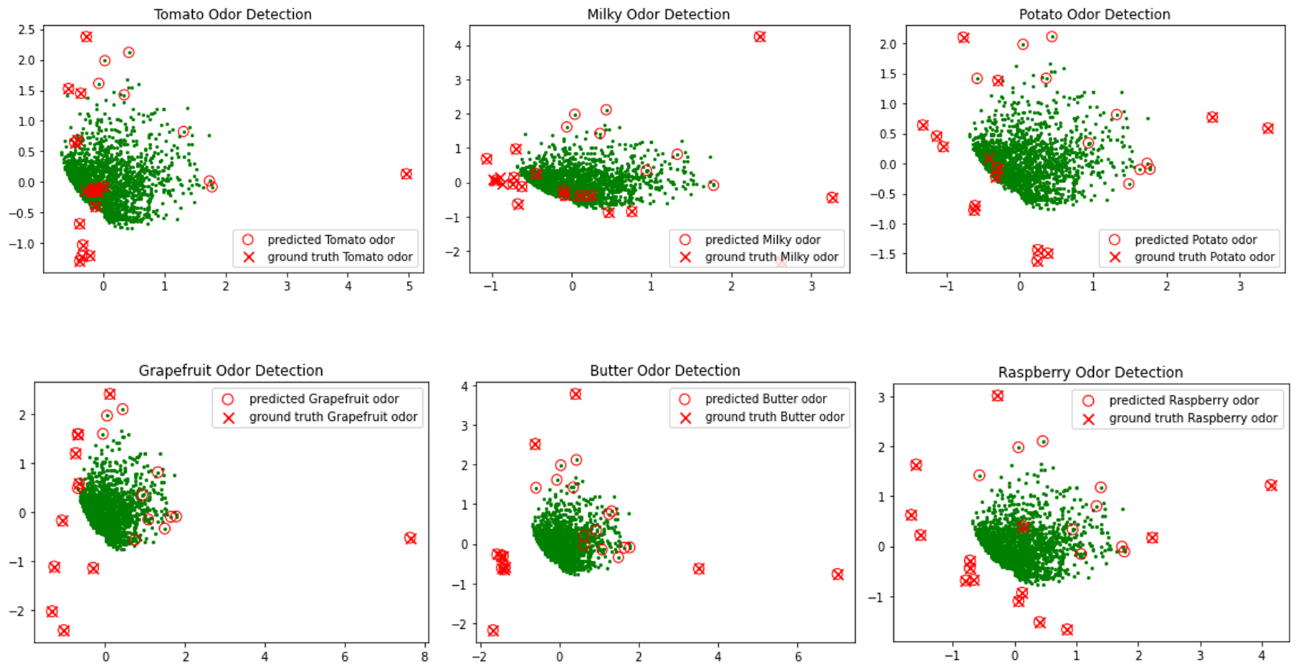


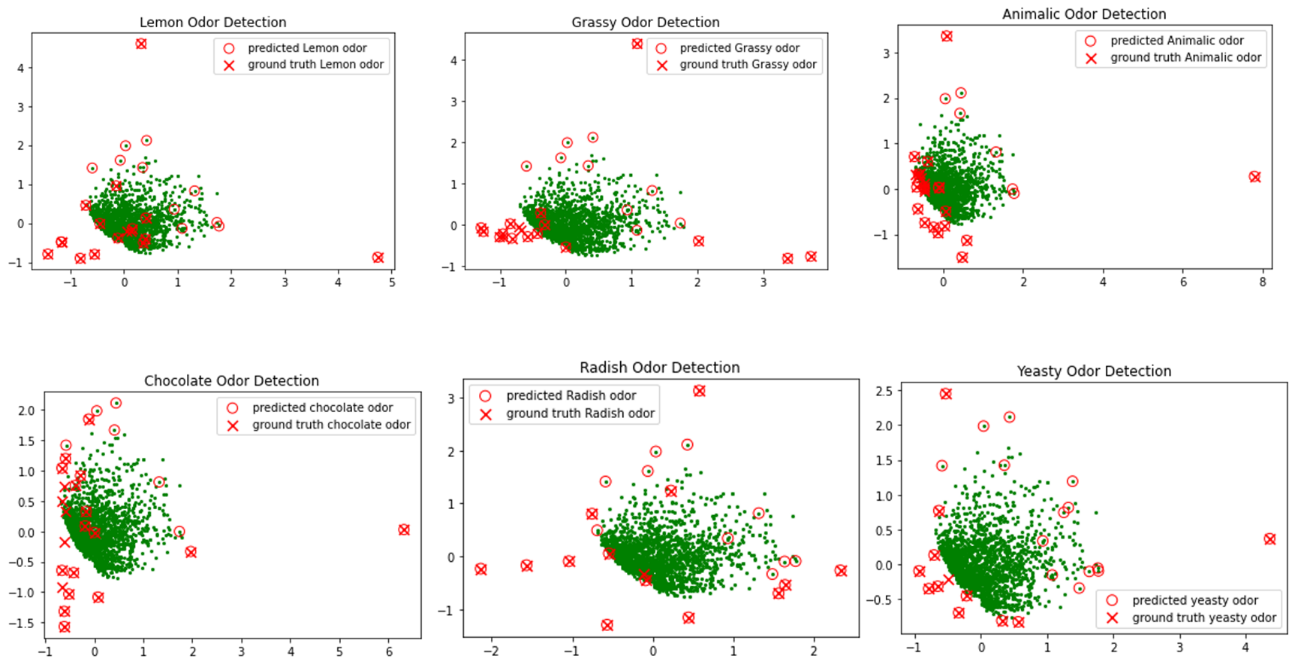


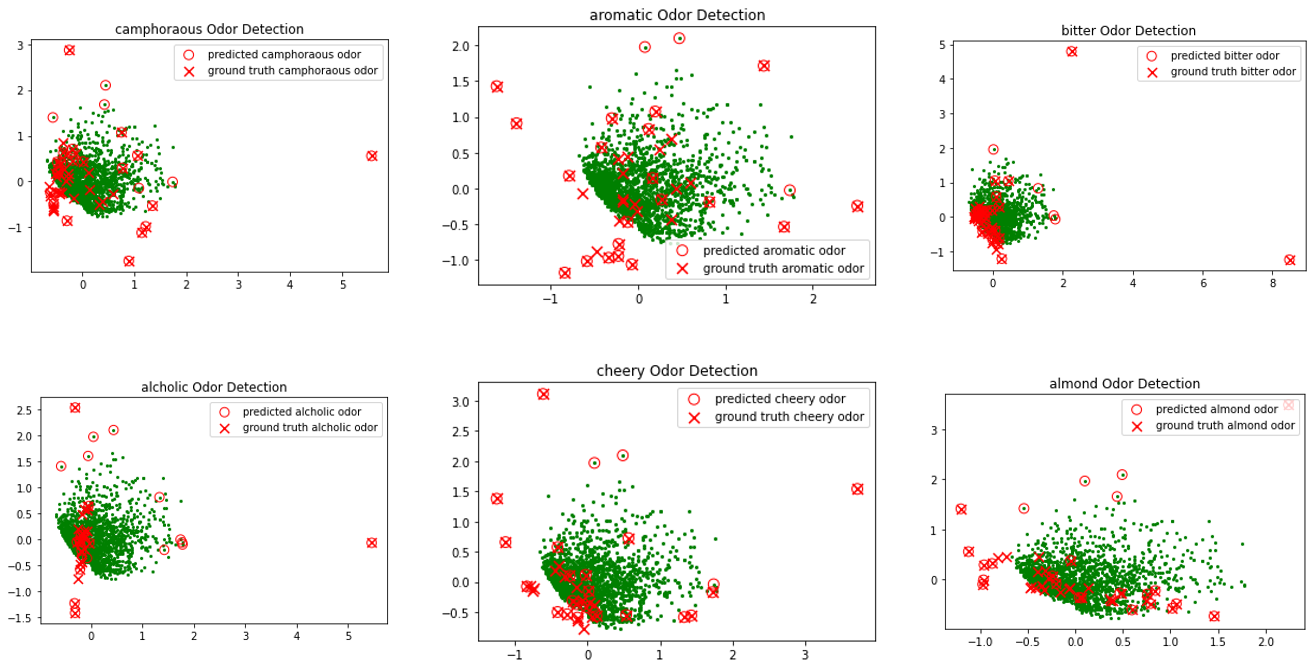


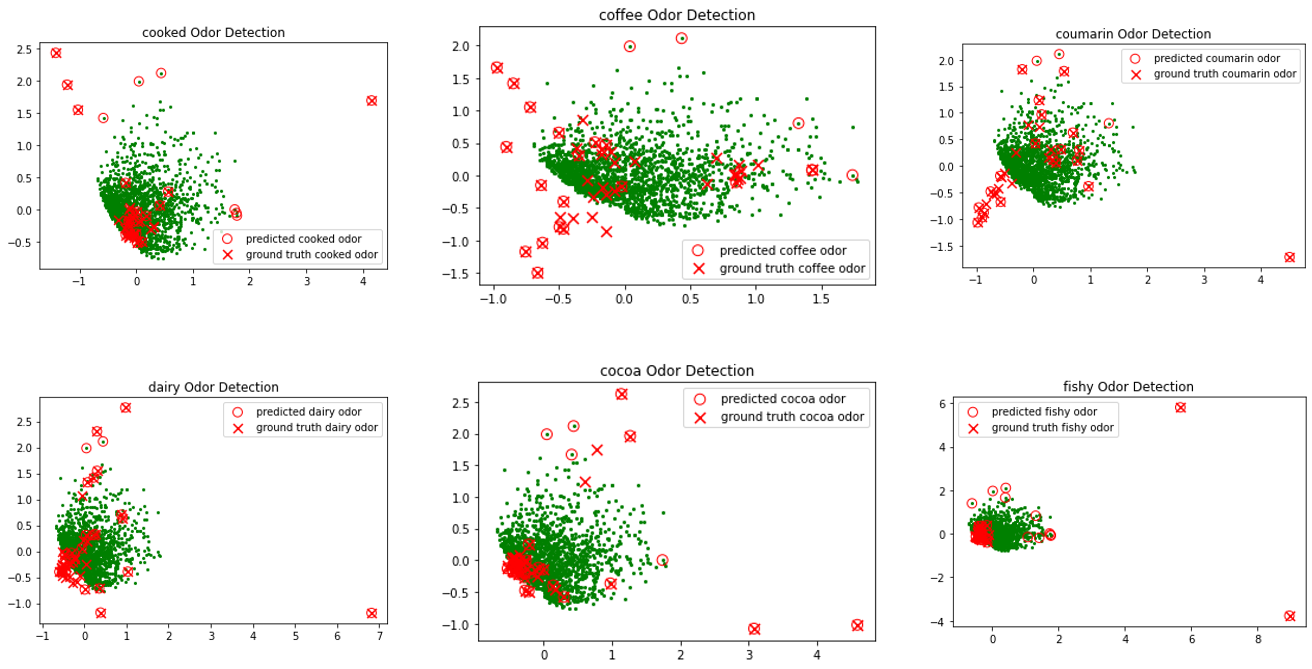


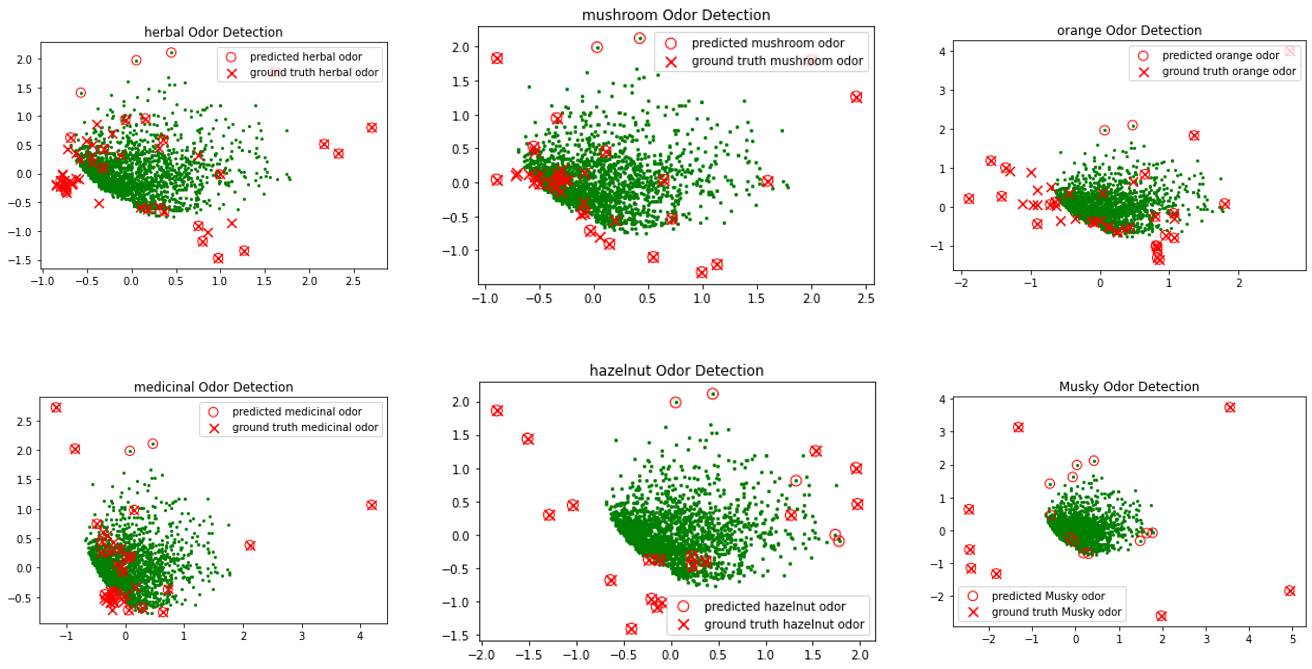


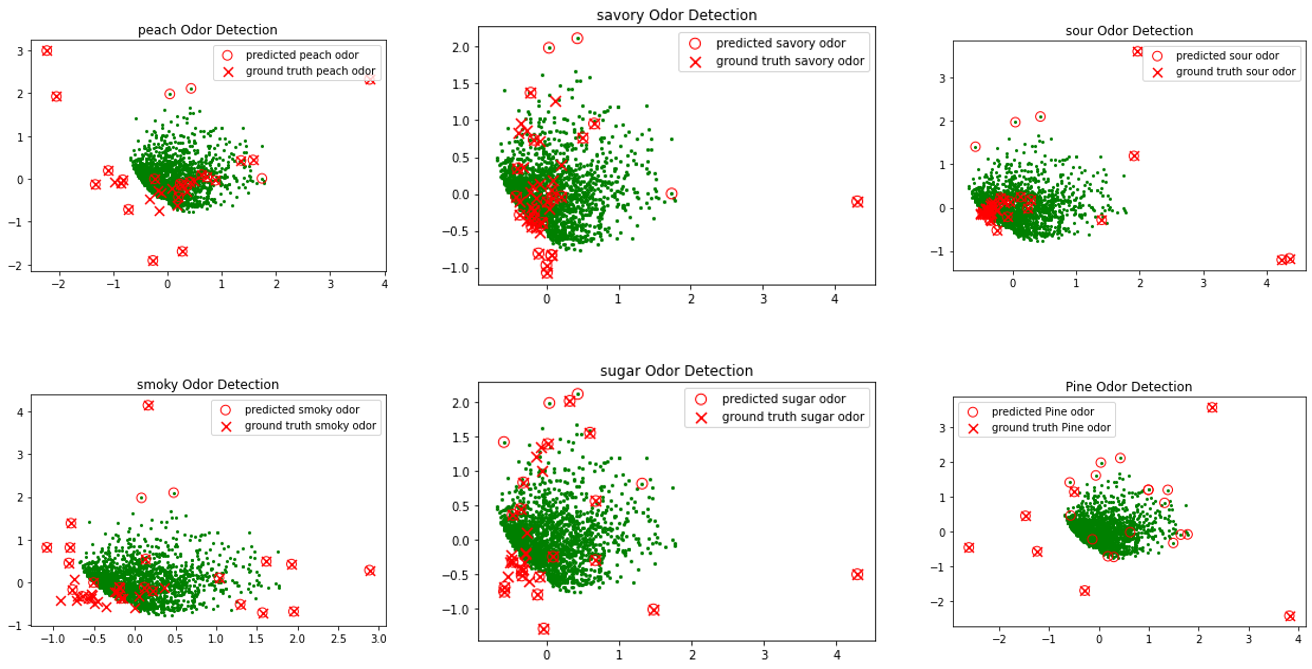


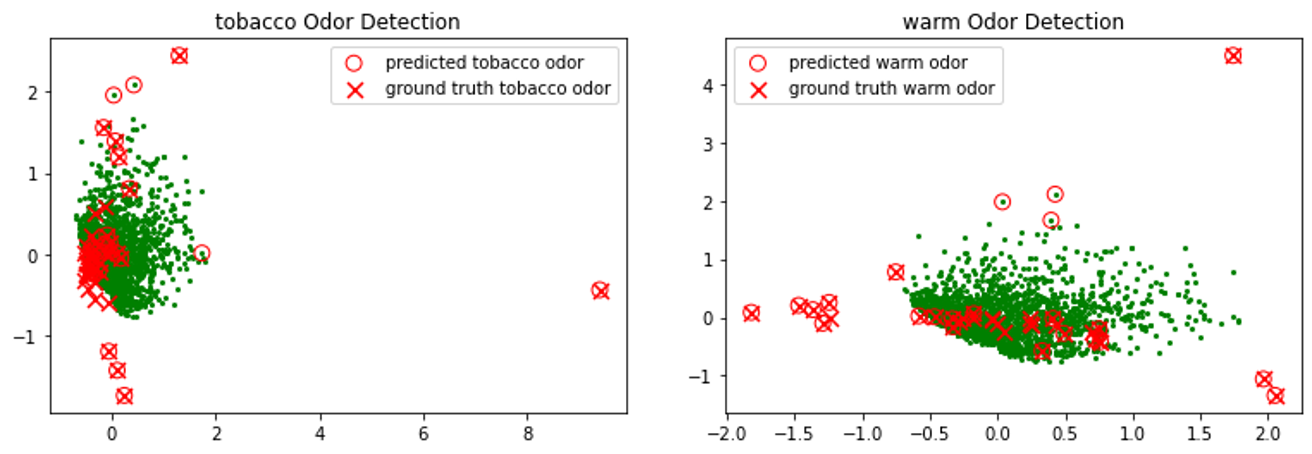

Supplement: Supplementary file 2 — Supplementary Information 2. [file 41598_2022_7802_MOESM2_ESM.docx]
