# Supplementary material for: Predicting individual perceptual scent impression from imbalanced dataset using mass spectrum of odorant molecules
Source: Sci Rep. 2022 Mar 8;12:3778. doi: 10.1038/s41598-022-07802-3 (PMC8904784; doi:10.1038/s41598-022-07802-3)
Supplement: Supplementary file 3 — Supplementary Information 3. [file 41598_2022_7802_MOESM3_ESM.docx]

Table S1: Large Category Odor descriptors

Optimized Hyper-parameters for each odor descriptors

| **Name of OD** | **C** | **gamma** |
| --- | --- | --- |
| Fruity | 10 | 1 |
| Green | 10 | 1 |
| Sweet | 10 | 1 |

Table S2: Middle Category Odor descriptors (Optimized Hyper-parameters)

| **Name of OD** | **C** | **gamma** |
| --- | --- | --- |
| apple | 10 | 1 |
| Banana | 10 | 1 |
| balsamic | 10 | 1 |
| berry | 10 | 1 |
| burnt | 10 | 1 |
| citrus | 10 | 1 |
| earthy | 10 | 1 |
| ethereal | 10 | 1 |
| fatty | 10 | 1 |
| fermented | 10 | 1 |
| floral | 10 | 1 |
| fresh | 10 | 1 |
| garlic | 0.001 | 1 |
| herbaceous | 10 | 1 |
| honey | 10 | 1 |
| meaty | 10 | 1 |
| melon | 10 | 1 |
| minty | 10 | 1 |
| musty | 10 | 1 |
| nutty | 10 | 1 |
| odorless | 10 | 1 |
| oily | 10 | 1 |
| onion | 10 | 1 |
| pear | 10 | 1 |
| phenolic | 10 | 1 |
| pineapple | 10 | 1 |
| pungent | 10 | 1 |
| roast | 10 | 1 |
| rose | 10 | 1 |
| spicy | 10 | 1 |
| sulfurous | 10 | 1 |
| tropical | 10 | 1 |
| waxy | 10 | 1 |
| winey | 10 | 1 |
| woody | 10 | 1 |
|  |  |  |

Table S3: Small Category Odor descriptors (Optimized Hyper-parameters)

| Name of OD | C | gamma |
| --- | --- | --- |
| Grape | 10 | 1 |
| Coconut | 0.001 | 1 |
| Anisic | 10 | 1 |
| cheesy | 0.001 | 1 |
| tea | 10 | 1 |
| cooling | 10 | 1 |
| strawberry | 0.001 | 1 |
| leafy | 0.001 | 1 |
| pleasant | 0.001 | 1 |
| Jasmine | 0.001 | 1 |
| Cinnamon | 0.001 | 1 |
| Cream | 0.001 | 1 |
| Tomato | 10 | 1 |
| Milky | 10 | 1 |
| Potato | 0.001 | 1 |
| grapefruit | 0.001 | 1 |
| Butter | 0.001 | 1 |
| Raspberry | 0.001 | 1 |
| lemon | 0.001 | 1 |
| Grassy | 10 | 1 |
| Animalic | 0.001 | 1 |
| Chocolate | 10 | 1 |
| Radish | 0.001 | 1 |
| Yeasty | 0.001 | 1 |
| pine | 0.001 | 1 |
| musky | 0.001 | 1 |
| hazelnut | 0.001 | 1 |
| peach | 0.001 | 1 |
| Spearmint | 0.001 | 1 |
| alcholic | 0.001 | 1 |
| almond | 10 | 1 |
| aromatic | 0.001 | 1 |
| bitter | 10 | 1 |
| camphoraous | 10 | 1 |
| cheery | 0.001 | 1 |
| cocoa | 10 | 1 |
| coffee | 10 | 1 |
| cooked | 0.001 | 1 |
| coumarin | 0.001 | 1 |
| dairy | 10 | 1 |
| fishy | 10 | 1 |
| herbal | 0.001 | 1 |
| medicinal | 10 | 1 |
| mushroom | 10 | 1 |
| orange | 10 | 1 |
| savory | 0.001 | 1 |
| smoky | 0.001 | 1 |
| sour | 10 | 1 |
| sugar | 0.001 | 1 |
| tobacco | 10 | 1 |
| warm | 0.001 | 1 |
