# Supplementary material for: Predicting individual perceptual scent impression from imbalanced dataset using mass spectrum of odorant molecules
Source: Sci Rep. 2022 Mar 8;12:3778. doi: 10.1038/s41598-022-07802-3 (PMC8904784; doi:10.1038/s41598-022-07802-3)
Supplement: Supplementary file 4 — Supplementary Information 4. [file 41598_2022_7802_MOESM4_ESM.docx]

Table S1: Middle Category odor descriptors using traditional MLP

| **Name of OD** | **TP** | **TN** | **FP** | **FN** | **ROC AUC** | **Recall** | **Target smell during testing** |
| --- | --- | --- | --- | --- | --- | --- | --- |
| apple | NA | 220 | NA | 15 | 0.839 | 0.000 | 15 |
| Banana | NA | 229 | NA | 6 | 0.872 | 0.000 | 6 |
| balsamic | NA | 216 | NA | 19 | 0.925 | 0.000 | 19 |
| berry | NA | 224 | NA | 11 | 0.448 | 0.000 | 11 |
| burnt | NA | 225 | NA | 10 | 0.759 | 0.000 | 10 |
| citrus | NA | 223 | NA | 12 | 0.922 | 0.000 | 12 |
| earthy | NA | 229 | NA | 6 | 0.617 | 0.000 | 6 |
| ethereal | NA | 225 | NA | 10 | 0.815 | 0.000 | 10 |
| fatty | 3 | 200 | 0 | 32 | 0.836 | 0.086 | 35 |
| fermented | NA | 230 | NA | 5 | 0.816 | 0.000 | 5 |
| floral | 1 | 192 | 1 | 41 | 0.771 | 0.024 | 42 |
| fresh | NA | 218 | NA | 17 | 0.623 | 0.000 | 17 |
| garlic | NA | 223 | NA | 12 | 0.800 | 0.000 | 12 |
| herbaceous | NA | 213 | NA | 22 | 0.530 | 0.000 | 22 |
| honey | NA | 227 | NA | 8 | 0.626 | 0.000 | 8 |
| meaty | NA | 221 | NA | 14 | 0.513 | 0.000 | 14 |
| melon | NA | 228 | NA | 7 | 0.665 | 0.000 | 7 |
| minty | NA | 230 | NA | 5 | 0.457 | 0.000 | 5 |
| musty | NA | 232 | NA | 3 | 0.448 | 0.000 | 3 |
| nutty | NA | 228 | NA | 7 | 0.767 | 0.000 | 7 |
| odorless | NA | 233 | NA | 2 | 0.582 | 0.000 | 2 |
| oily | NA | 227 | NA | 8 | 0.695 | 0.000 | 8 |
| onion | NA | 223 | NA | 12 | 0.854 | 0.000 | 12 |
| pear | NA | 233 | NA | 2 | 0.867 | 0.000 | 2 |
| phenolic | NA | 227 | NA | 8 | 0.917 | 0.000 | 8 |
| pineapple | NA | 223 | NA | 12 | 0.858 | 0.000 | 12 |
| pungent | NA | 225 | NA | 10 | 0.711 | 0.000 | 10 |
| roast | NA | 224 | NA | 11 | 0.749 | 0.000 | 11 |
| rose | NA | 224 | NA | 11 | 0.732 | 0.000 | 11 |
| spicy | NA | 217 | NA | 18 | 0.848 | 0.000 | 18 |
| sulfurous | 2 | 220 | 0 | 13 | 0.828 | 0.133 | 15 |
| tropical | NA | 221 | NA | 14 | 0.798 | 0.000 | 14 |
| waxy | NA | 216 | NA | 19 | 0.862 | 0.000 | 19 |
| winey | NA | 230 | NA | 5 | 0.700 | 0.000 | 5 |
| woody | NA | 217 | NA | 18 | 0.701 | 0.000 | 18 |

Table S2: Small Category odor descriptors using traditional MLP

| **Name of OD** | **TP** | **TN** | **FP** | **FN** | **ROC AUC** | **Recall** | **Target smell during testing** |
| --- | --- | --- | --- | --- | --- | --- | --- |
| Grape | NA | 232 | NA | 3 | 0.995 | 0.000 | 3 |
| Coconut | NA | 232 | NA | 3 | 0.748 | 0.000 | 3 |
| Anisic | 0 | 230 | 1 | 4 | 0.825 | 0.000 | 4 |
| cheesy | NA | 231 | NA | 4 | 0.223 | 0.000 | 4 |
| tea | 3 | 131 | 99 | 2 | 0.448 | 0.600 | 5 |
| cooling | NA | 233 | NA | 2 | 0.283 | 0.000 | 2 |
| strawberry | NA | 233 | NA | 2 | 0.405 | 0.000 | 2 |
| leafy | NA | 233 | NA | 2 | 0.804 | 0.000 | 2 |
| pleasant | NA | 234 | NA | 1 | 0.603 | 0.000 | 1 |
| Jasmine | NA | 232 | NA | 3 | 0.599 | 0.000 | 3 |
| Cinnamon | NA | 230 | 2 | 3 | 0.328 | 0.000 | 3 |
| Cream | NA | 233 | NA | 2 | 0.778 | 0.000 | 2 |
| Tomato | NA | 234 | NA | 1 | 0.669 | 0.000 | 1 |
| Milky | 1 | 32 | 202 | 0 | 0.276 | 1.000 | 1 |
| Potato | 1 | 1 | 233 | 0 | 0.457 | 1.000 | 1 |
| grapefruit | NA | 232 | NA | 3 | 0.847 | 0.000 | 3 |
| Butter | NA | 233 | NA | 2 | 0.597 | 0.000 | 2 |
| Raspberry | NA | NA | NA | NA | NA | NA | NA |
| lemon | NA | 233 | NA | 2 | 0.959 | 0.000 | 2 |
| Grassy | NA | 232 | NA | 3 | 0.726 | 0.000 | 3 |
| Animalic | NA | 234 | NA | 1 | 0.994 | 0.000 | 1 |
| Chocolate | 1 | 89 | 145 | 0 | 0.936 | 0.000 | 1 |
| Radish | NA | 233 | NA | 2 | 0.365 | 0.000 | 2 |
| Yeasty | NA | 234 | NA | 1 | 0.165 | 0.000 | 1 |
| pine | NA | 220 | 13 | 2 | 0.472 | 0.000 | 2 |
| musky | NA | 233 | NA | 2 | 0.798 | 0.000 | 2 |
| hazelnut | NA | 234 | NA | 1 | 0.912 | 0.000 | 1 |
| peach | NA | 231 | NA | 4 | 0.569 | 0.000 | 4 |
| Spearmint | NA | 234 | NA | 1 | 0.838 | 0.000 | 1 |
| alcoholic | NA | 234 | NA | 1 | 0.936 | 0.000 | 1 |
| almond | NA | 229 | NA | 6 | 0.955 | 0.000 | 6 |
| aromatic | NA | 232 | NA | 3 | 0.702 | 0.000 | 3 |
| bitter | NA | 226 | NA | 9 | 0.635 | 0.000 | 9 |
| camphoraous | NA | 182 | 50 | 3 | 0.518 | 0.000 | 3 |
| cheery | NA | 231 | NA | 4 | 0.143 | 0.000 | 4 |
| cocoa | NA | 232 | NA | 3 | 0.606 | 0.000 | 3 |
| coffee | NA | 232 | NA | 3 | 0.877 | 0.000 | 3 |
| cooked | NA | 230 | NA | 5 | 0.620 | 0.000 | 5 |
| coumarin | NA | 231 | NA | 4 | 0.646 | 0.000 | 4 |
| dairy | NA | 230 | NA | 5 | 0.691 | 0.000 | 5 |
| fishy | NA | 232 | NA | 3 | 0.911 | 0.000 | 3 |
| herbal | NA | 228 | NA | 7 | 0.573 | 0.000 | 7 |
| medicinal | NA | 230 | NA | 5 | 0.733 | 0.000 | 5 |
| mushroom | NA | 233 | NA | 2 | 0.631 | 0.000 | 2 |
| orange | NA | 231 | NA | 4 | 0.598 | 0.000 | 4 |
| savory | 5 | 37 | 192 | 1 | 0.240 | 0.833 | 6 |
| smoky | NA | 228 | NA | 7 | 0.862 | 0.000 | 7 |
| sour | NA | 227 | NA | 8 | 0.657 | 0.000 | 8 |
| sugar | 1 | 188 | 44 | 2 | 0.632 | 0.333 | 3 |
| tobacco | NA | 233 | NA | 2 | 0.793 | 0.000 | 2 |
| warm | 7 | 24 | 204 | 0 | 0.578 | 1.000 | 7 |

Table S3: Middle Category odor descriptors using Cost sensitive MLP

| **Name of OD** | **TP** | **TN** | **FP** | **FN** | **ROC AUC** | **Recall** | **Target smell during testing** | **Weight for class 0** | **Weight for class 1** |
| --- | --- | --- | --- | --- | --- | --- | --- | --- | --- |
| apple | 13 | 128 | 92 | 2 | 0.835 | 0.867 | 15 | 0.54 | 6.74 |
| Banana | 5 | 185 | 44 | 1 | 0.912 | 0.833 | 6 | 0.51 | 17.24 |
| balsamic | 17 | 176 | 40 | 2 | 0.927 | 0.895 | 19 | 0.53 | 9.16 |
| berry | 4 | 163 | 61 | 7 | 0.494 | 0.364 | 11 | 0.52 | 15.84 |
| burnt | 9 | 121 | 104 | 1 | 0.726 | 0.900 | 10 | 0.52 | 11.27 |
| citrus | 12 | 177 | 46 | 0 | 0.931 | 1.000 | 12 | 0.53 | 9.61 |
| earthy | 0 | 201 | 28 | 6 | 0.559 | 0.000 | 6 | 0.53 | 9.46 |
| ethereal | 8 | 161 | 64 | 2 | 0.866 | 0.800 | 10 | 0.53 | 9.02 |
| fatty | 28 | 162 | 38 | 7 | 0.835 | 0.800 | 35 | 0.57 | 4.26 |
| fermented | 4 | 131 | 99 | 1 | 0.838 | 0.800 | 5 | 0.52 | 16.75 |
| floral | 26 | 153 | 40 | 16 | 0.784 | 0.619 | 42 | 0.59 | 3.39 |
| fresh | 9 | 156 | 62 | 8 | 0.634 | 0.529 | 17 | 0.53 | 9.46 |
| garlic | 10 | 178 | 45 | 2 | 0.891 | 0.833 | 12 | 0.51 | 21.32 |
| herbaceous | 11 | 129 | 84 | 11 | 0.605 | 0.500 | 22 | 0.55 | 5.83 |
| honey | 3 | 191 | 36 | 5 | 0.618 | 0.375 | 8 | 0.52 | 16.28 |
| meaty | NA | 221 | NA | 14 | 0.582 | 0.000 | 14 | 0.52 | 13.63 |
| melon | 4 | 177 | 51 | 3 | 0.607 | 0.571 | 7 | 0.51 | 22.55 |
| minty | NA | 230 | NA | 5 | 0.457 | 0.000 | 5 | 0.52 | 15.43 |
| musty | NA | 232 | NA | 3 | 0.448 | 0.000 | 3 | 0.52 | 16.28 |
| nutty | 6 | 75 | 153 | 1 | 0.741 | 0.857 | 7 | 0.53 | 8.88 |
| odorless | NA | 179 | 54 | 2 | 0.601 | 0.000 | 2 | 0.51 | 19.87 |
| oily | 4 | 168 | 59 | 4 | 0.678 | 0.500 | 8 | 0.53 | 8.5 |
| onion | 10 | 183 | 40 | 2 | 0.865 | 0.833 | 12 | 0.52 | 15.63 |
| pear | 2 | 181 | 52 | 0 | 0.946 | 1.000 | 2 | 0.52 | 16.99 |
| phenolic | 7 | 194 | 33 | 1 | 0.931 | 0.875 | 8 | 0.51 | 17.5 |
| pineapple | 8 | 185 | 38 | 4 | 0.865 | 0.667 | 12 | 0.52 | 11.27 |
| pungent | 6 | 163 | 62 | 4 | 0.756 | 0.600 | 10 | 0.52 | 12.88 |
| roast | 8 | 148 | 76 | 3 | 0.764 | 0.727 | 11 | 0.52 | 11.06 |
| rose | 5 | 188 | 36 | 6 | 0.722 | 0.455 | 11 | 0.52 | 15.03 |
| spicy | 16 | 161 | 56 | 2 | 0.876 | 0.889 | 18 | 0.53 | 7.76 |
| sulfurous | 11 | 171 | 49 | 4 | 0.836 | 0.733 | 15 | 0.53 | 8.69 |
| tropical | 11 | 159 | 62 | 3 | 0.791 | 0.786 | 14 | 0.53 | 9.46 |
| waxy | 17 | 170 | 46 | 2 | 0.888 | 0.895 | 19 | 0.54 | 6.2 |
| winey | 4 | 151 | 79 | 1 | 0.694 | 0.800 | 5 | 0.53 | 9.23 |
| woody | 9 | 177 | 40 | 9 | 0.724 | 0.500 | 18 | 0.54 | 7.56 |

Table S4: Small Category odor descriptors using Cost sensitive MLP

| **Name of**  **OD** | **TP** | **TN** | **FP** | **FN** | **ROC AUC** | **Recall** | **Target smell during testing** | **Weight for class 0** | **Weight for class 1** |
| --- | --- | --- | --- | --- | --- | --- | --- | --- | --- |
| Grape | 3 | 163 | 69 | 0 | 0.993 | 1.000 | 3 | 0.5 | 106.59 |
| Coconut | 1 | 174 | 58 | 2 | 0.511 | 0.333 | 3 | 0.51 | 40.43 |
| Anisic | 3 | 184 | 47 | 1 | 0.910 | 0.750 | 4 | 0.51 | 41.88 |
| cheesy | NA | 231 | NA | 4 | 0.223 | 0.000 | 4 | 0.51 | 40.43 |
| tea | 3 | 79 | 151 | 2 | 0.557 | 0.600 | 5 | 0.51 | 46.9 |
| cooling | NA | 233 | NA | 2 | 0.283 | 0.000 | 2 | 0.5 | 90.19 |
| strawberry | NA | 233 | 2 | 2 | 0.405 | 0.000 | 2 | 0.5 | 68.97 |
| leafy | 1 | 157 | 76 | 1 | 0.829 | 0.500 | 2 | 0.51 | 41.88 |
| pleasant | NA | 234 | NA | 1 | 0.603 | 0.000 | 1 | 0.5 | 53.3 |
| Jasmine | 232 | 232 | 3 | 3 | 0.599 |  | 3 | 0.5 | 97.71 |
| Cinnamon | NA | 178 | 54 | 3 | 0.336 | 0.000 | 3 | 0.5 | 65.14 |
| Cream | NA | 233 | NA | 2 | 0.778 | 0.000 | 2 | 0.5 | 65.14 |
| Tomato | NA | 234 | NA | 1 | 0.669 | 0.000 | 1 | 0.5 | 58.62 |
| Milky | 1 | 171 | 63 | 0 | 0.991 | 1.000 | 1 | 0.5 | 55.83 |
| Potato | 1 | 13 | 221 | 0 | 0.483 | 1.000 | 1 | 0.5 | 78.17 |
| grapefruit | 1 | 211 | 21 | 2 | 0.843 | 0.333 | 3 | 0.5 | 106.59 |
| Butter | NA | 233 | NA | 2 | 0.597 | 0.000 | 2 | 0.5 | 106.59 |
| Raspberry | NA | NA | NA | NA | NA | NA | NA | 0.5 | 78.17 |
| lemon | 2 | 211 | 22 | 0 | 0.956 | 1.000 | 2 | 0.5 | 68.97 |
| Grassy | 1 | 175 | 57 | 2 | 0.642 | 0.333 | 3 | 0.5 | 65.14 |
| Animalic | 1 | 176 | 58 | 0 | 0.996 | 1.000 | 1 | 0.51 | 46.9 |
| Chocolate | 1 | 176 | 58 | 0 | 1.000 | 1.000 | 1 | 0.5 | 55.83 |
| Radish | NA | 196 | 37 | 2 | 0.464 | 0.000 | 2 | 0.5 | 83.75 |
| Yeasty | NA | 234 |  | 1 | 0.165 | 0.000 | 1 | 0.5 | 97.71 |
| pine | NA | 205 | 28 | 2 | 0.570 | 0.000 | 2 | 0.5 | 167.5 |
| musky | NA | 227 | 6 | 2 | 0.768 | 0.000 | 2 | 0.5 | 146.56 |
| hazelnut | 1 | 174 | 60 | 0 | 0.893 | 1.000 | 1 | 0.5 | 65.14 |
| peach | 1 | 196 | 35 | 3 | 0.535 | 0.250 | 4 | 0.51 | 40.43 |
| Spearmint | 0 | 215 | 19 | 1 | 0.795 | 0.000 | 1 | 0.5 | 83.75 |
| alcoholic | 1 | 209 | 25 | 0 | 0.976 | 1.000 | 1 | 0.51 | 37.82 |
| almond | 6 | 185 | 44 | 0 | 0.947 | 1.000 | 6 | 0.51 | 29.31 |
| aromatic | 1 | 199 | 33 | 2 | 0.658 | 0.333 | 3 | 0.51 | 33.5 |
| bitter | 4 | 178 | 48 | 5 | 0.712 | 0.444 | 9 | 0.51 | 23.93 |
| camphoraous | 2 | 84 | 148 | 1 | 0.506 | 0.667 | 3 | 0.51 | 24.43 |
| cheery | 3 | 195 | 36 | 1 | 0.930 | 0.750 | 4 | 0.51 | 34.49 |
| cocoa | 2 | 190 | 42 | 1 | 0.688 | 0.667 | 3 | 0.51 | 24.95 |
| coffee | 2 | 119 | 113 | 1 | 0.758 | 0.667 | 3 | 0.51 | 26.06 |
| cooked | 2 | 185 | 45 | 3 | 0.723 | 0.400 | 5 | 0.51 | 30.86 |
| coumarin | 4 | 107 | 124 | 0 | 0.668 | 1.000 | 4 | 0.51 | 36.64 |
| dairy | 5 | 176 | 54 | 0 | 0.871 | 1.000 | 5 | 0.51 | 23.45 |
| fishy | 3 | 199 | 33 | 0 | 0.938 | 1.000 | 3 | 0.51 | 29.31 |
| herbal | 1 | 183 | 45 | 6 | 0.489 | 0.143 | 7 | 0.51 | 23.45 |
| medicinal | 3 | 194 | 36 | 2 | 0.793 | 0.600 | 5 | 0.51 | 30.06 |
| mushroom | NA | 207 | 26 | 2 | 0.514 | 0.000 | 2 | 0.51 | 30.86 |
| orange | NA | 200 | 31 | 4 | 0.701 | 0.000 | 4 | 0.51 | 27.27 |
| savory | 3 | 177 | 52 | 3 | 0.660 | 0.500 | 6 | 0.51 | 26.65 |
| smoky | 6 | 195 | 33 | 1 | 0.904 | 0.857 | 7 | 0.51 | 30.06 |
| sour | 3 | 219 | 8 | 5 | 0.708 | 0.375 | 8 | 0.51 | 23.93 |
| sugar | 3 | 150 | 82 | 0 | 0.910 | 1.000 | 3 | 0.51 | 35.53 |
| tobacco | 2 | 152 | 81 | 0 | 0.752 | 1.000 | 2 | 0.51 | 26.06 |
| warm | 7 | 36 | 192 | 0 | 0.582 | 1.000 | 7 | 0.51 | 34.49 |
