# Supplementary material for: Predicting individual perceptual scent impression from imbalanced dataset using mass spectrum of odorant molecules
Source: Sci Rep. 2022 Mar 8;12:3778. doi: 10.1038/s41598-022-07802-3 (PMC8904784; doi:10.1038/s41598-022-07802-3)
Supplement: Supplementary file 5 — Supplementary Information 5. [file 41598_2022_7802_MOESM5_ESM.docx]

This table is for statistical analysis on small category dataset using OCSVM where 2^nd^ column indicates the 5-fold cross-validation performance when we used positive samples for training and 3^rd^ column where we used negative samples for training.

| **Name of OD** | **Training with positive samples** | **Training with negative samples** |
| --- | --- | --- |
| Grape | 0.623 | 0.990 |
| Coconut | 0.639 | 0.995 |
| Anisic | 0.633 | 0.999 |
| cheesy | 0.630 | 0.993 |
| tea | 0.588 | 0.999 |
| cooling | 0.570 | 1.000 |
| strawberry | 0.509 | 0.999 |
| leafy | 0.638 | 0.993 |
| pleasant | 0.548 | 0.999 |
| Jasmine | 0.558 | 1.000 |
| Cinnamon | 0.463 | 1.000 |
| Cream | 0.468 | 0.999 |
| Tomato | 0.494 | 0.960 |
| Milky | 0.546 | 0.999 |
| Potato | 0.584 | 0.993 |
| grapefruit | 0.524 | 1.000 |
| Butter | 0.524 | 1.000 |
| Raspberry | 0.584 | 1.000 |
| lemon | 0.512 | 0.999 |
| Grassy | 0.476 | 0.999 |
| Animalic | 0.601 | 0.994 |
| Chocolate | 0.551 | 0.999 |
| Radish | 0.559 | 0.999 |
| Yeasty | 0.560 | 0.997 |
| pine | 0.466 | 1.000 |
| musky | 0.531 | 1.000 |
| hazelnut | 0.468 | 1.000 |
| peach | 0.627 | 0.998 |
| Spearmint | 0.567 | 0.999 |
| alcoholic | 0.729 | 0.799 |
| almond | 0.661 | 0.952 |
| aromatic | 0.671 | 0.971 |
| bitter | 0.689 | 0.955 |
| camphoraous | 0.683 | 0.968 |
| cheery | 0.683 | 0.977 |
| cocoa | 0.696 | 0.943 |
| coffee | 0.667 | 0.787 |
| cooked | 0.672 | 0.864 |
| coumarin | 0.667 | 0.976 |
| dairy | 0.690 | 0.972 |
| fishy | 0.663 | 0.895 |
| herbal | 0.723 | 0.940 |
| medicinal | 0.664 | 0.995 |
| mushroom | 0.672 | 0.905 |
| orange | 0.667 | 0.989 |
| savory | 0.657 | 0.836 |
| smoky | 0.676 | 0.997 |
| sour | 0.692 | 0.949 |
| sugar | 0.664 | 0.978 |
| tobacco | 0.671 | 0.987 |
| warm | 0.658 | 0.935 |
