# Supplementary material for: Predicting individual perceptual scent impression from imbalanced dataset using mass spectrum of odorant molecules
Source: Sci Rep. 2022 Mar 8;12:3778. doi: 10.1038/s41598-022-07802-3 (PMC8904784; doi:10.1038/s41598-022-07802-3)
Supplement: Supplementary file 6 — Supplementary Information 6. [file 41598_2022_7802_MOESM6_ESM.docx]

Comparison between GNN model (reference 8) and this study (mainly with CSMLP and OCSVM) for each odor descriptor.

**Table S1:** large Category Odor Descriptors

| **Name of OD** | **GNN [8]** | **CSMLP** | **OCSVM** | **Traditional MLP** | **SVM** |
| --- | --- | --- | --- | --- | --- |
| Fruity | 0.859 | 0.76 | 0.55 | 0.75 | 0.691 |
| Green | 0.818 | 0.7 | 0.54 | 0.7 | 0.598 |
| Sweet | 0.747 | 0.698 | 0.53 | 0.69 | 0.613 |

**Table S2**: Middle category Odor Descriptors

| **Name of OD** | **GNN [8]** | **CSMLP** | **OCSVM** | **Traditional MLP** | **SVM** |
| --- | --- | --- | --- | --- | --- |
| apple | 0.917 | 0.83 | 0.746 | 0.83 | 0.682 |
| Banana | 0.961 | 0.9 | 0.743 | 0.895 | 0.766 |
| balsamic | 0.91 | 0.888 | 0.915 | 0.867 | 0.780 |
| berry | 0.858 | 0.694 | 0.911 | 0.621 | 0.556 |
| burnt | 0.903 | 0.817 | 0.648 | 0.809 | 0.670 |
| citrus | 0.918 | 0.88 | 0.858 | 0.873 | 0.649 |
| earthy | 0.745 | 0.692 | 0.668 | 0.675 | 0.603 |
| ethereal | 0.915 | 0.864 | 0.529 | 0.846 | 0.752 |
| fatty | 0.898 | 0.867 | 0.807 | 0.873 | 0.799 |
| fermented | 0.895 | 0.813 | 0.748 | 0.8 | 0.664 |
| floral | 0.852 | 0.796 | 0.673 | 0.803 | 0.713 |
| fresh | 0.756 | 0.777 | 0.727 | 0.751 | 0.618 |
| garlic | 0.986 | 0.902 | 0.772 | 0.85 | 0.561 |
| herbaceous | NA | 0.718 | 0.676 | 0.692 | 0.597 |
| honey | 0.872 | 0.902 | 0.897 | 0.854 | 0.686 |
| meaty | 0.945 | 0.831 | 0.704 | 0.733 | 0.800 |
| melon | 0.884 | 0.803 | 0.871 | 0.763 | 0.617 |
| minty | 0.898 | 0.827 | 0.935 | 0.818 | 0.727 |
| musty | 0.774 | 0.695 | 0.849 | 0.604 | 0.537 |
| nutty | 0.844 | 0.819 | 0.654 | 0.799 | 0.710 |
| odorless | 0.973 | 0.859 | 0.951 | 0.832 | 0.726 |
| oily | 0.833 | 0.854 | 0.892 | 0.849 | 0.781 |
| onion | 0.979 | 0.898 | 0.685 | 0.88 | 0.822 |
| pear | 0.952 | 0.854 | 0.878 | 0.847 | 0.695 |
| phenolic | 0.949 | 0.937 | 0.928 | 0.927 | 0.830 |
| pineapple | 0.954 | 0.838 | 0.795 | 0.829 | 0.755 |
| pungent | 0.886 | 0.795 | 0.678 | 0.8 | 0.695 |
| roast | 0.932 | 0.797 | 0.614 | 0.8 | 0.682 |
| rose | 0.92 | 0.88 | 0.830 | 0.865 | 0.670 |
| spicy | 0.813 | 0.851 | 0.762 | 0.853 | 0.748 |
| sulfurous | 0.983 | 0.85 | 0.535 | 0.861 | 0.810 |
| tropical | 0.848 | 0.811 | 0.749 | 0.813 | 0.635 |
| waxy | 0.902 | 0.891 | 0.838 | 0.891 | 0.772 |
| winey | 0.902 | 0.827 | 0.615 | 0.805 | 0.655 |
| woody | 0.873 | 0.841 | 0.835 | 0.847 | 0.729 |

**Table S3:** Small Category Odor Descriptors

| **Name of OD** | **GNN [8]** | **CSMLP** | **OCSVM** | **Traditional MLP** | **SVM** |
| --- | --- | --- | --- | --- | --- |
| Grape | 0.953 | 0.922 | 0.99 | 0.893 | 0.771 |
| Coconut | 0.959 | 0.828 | 0.995 | 0.811 | 0.452 |
| Anisic | 0.791 | 0.917 | 0.999 | 0.907 | 0.726 |
| cheesy | 0.92 | 0.888 | 0.993 | 0.871 | 0.642 |
| tea | 0.745 | 0.821 | 0.999 | 0.757 | 0.476 |
| cooling | 0.973 | 0.911 | 1 | 0.833 | 0.803 |
| strawberry | 0.909 | 0.755 | 0.999 | 0.733 | 0.468 |
| leafy | 0.842 | 0.756 | 0.993 | 0.751 | 0.369 |
| pleasant | NA | 0.758 | 0.999 | 0.671 | 0.663 |
| Jasmine | 0.951 | 0.849 | 1 | 0.804 | 0.472 |
| Cinnamon | 0.88 | 0.937 | 1 | 0.925 | 0.676 |
| Cream | 0.808 | 0.763 | 0.999 | 0.598 | 0.541 |
| Tomato | 0.941 | 0.873 | 0.96 | 0.841 | 0.587 |
| Milky | 0.849 | 0.79 | 0.999 | 0.689 | 0.588 |
| Potato | 0.983 | 0.891 | 0.993 | 0.847 | 0.550 |
| grapefruit | 0.929 | 0.917 | 1 | 0.887 | 0.505 |
| Butter | 0.881 | 0.944 | 1 | 0.906 | 0.378 |
| Raspberry | 0.827 | 0.81 | 1 | 0.829 | 0.582 |
| lemon | 0.855 | 0.952 | 0.999 | 0.94 | 0.570 |
| Grassy | 0.845 | 0.863 | 0.999 | 0.803 | 0.538 |
| Animalic | 0.812 | 0.845 | 0.994 | 0.835 | 0.537 |
| Chocolate | 0.925 | 0.827 | 0.999 | 0.782 | 0.683 |
| Radish | 0.923 | 0.896 | 0.999 | 0.792 | 0.603 |
| Yeasty | NA | 0.858 | 0.997 | 0.835 | 0.597 |
| pine | 0.956 | 0.989 | 1 | 0.983 | 0.513 |
| musky | 0.917 | 0.809 | 1 | 0.759 | 0.550 |
| hazelnut | 0.987 | 0.917 | 1 | 0.905 | 0.594 |
| peach | 0.839 | 0.868 | 0.9985 | 0.727 | 0.514 |
| Spearmint | NA | 0.881 | 0.999 | 0.857 | 0.671 |
| alcholic | 0.961 | 0.954 | 0.7985 | 0.917 | 0.481 |
| almond | 0.943 | 0.924 | 0.9518 | 0.893 | 0.683 |
| aromatic | 0.855 | 0.852 | 0.9707 | 0.818 | 0.547 |
| bitter | 0.719 | 0.791 | 0.9552 | 0.734 | 0.545 |
| camphoraous | 0.951 | 0.862 | 0.968 | 0.836 | 0.703 |
| cheery | 0.905 | 0.91 | 0.9766 | 0.92 | 0.344 |
| cocoa | 0.938 | 0.824 | 0.9434 | 0.811 | 0.692 |
| coffee | 0.938 | 0.873 | 0.7866 | 0.841 | 0.761 |
| cooked | 0.848 | 0.768 | 0.864 | 0.685 | 0.487 |
| coumarin | 0.95 | 0.851 | 0.9757 | 0.826 | 0.634 |
| dairy | 0.884 | 0.828 | 0.9723 | 0.779 | 0.664 |
| fishy | 0.915 | 0.886 | 0.8954 | 0.87 | 0.810 |
| herbal | 0.766 | 0.7 | 0.9399 | 0.631 | 0.510 |
| medicinal | 0.906 | 0.888 | 0.9955 | 0.861 | 0.722 |
| mushroom | 0.91 | 0.745 | 0.9049 | 0.635 | 0.580 |
| orange | 0.933 | 0.843 | 0.9888 | 0.797 | 0.637 |
| savory | 0.944 | 0.767 | 0.8358 | 0.701 | 0.490 |
| smoky | 0.909 | 0.934 | 0.9969 | 0.908 | 0.609 |
| sour | 0.839 | 0.851 | 0.9492 | 0.781 | 0.731 |
| sugar | NA | 0.883 | 0.9776 | 0.871 | 0.507 |
| tobacco | 0.877 | 0.79 | 0.9875 | 0.786 | 0.475 |
| warm | 0.815 | 0.73 | 0.9353 | 0.667 | 0.501 |
